# GATA3 induces human T-cell commitment by restraining Notch activity and repressing NK-cell fate

**DOI:** 10.1038/ncomms11171

**Published:** 2016-04-06

**Authors:** Inge Van de Walle, Anne-Catherine Dolens, Kaat Durinck, Katrien De Mulder, Wouter Van Loocke, Sagar Damle, Els Waegemans, Jelle De Medts, Imke Velghe, Magda De Smedt, Bart Vandekerckhove, Tessa Kerre, Jean Plum, Georges Leclercq, Ellen V. Rothenberg, Pieter Van Vlierberghe, Frank Speleman, Tom Taghon

**Affiliations:** 1 Faculty of Medicine and Health Sciences, Department of Clinical Chemistry, Microbiology and Immunology, Ghent University, University Hospital Ghent, 4BlokA, De Pintelaan 185, B-9000 Ghent, Belgium; 2 Center for Medical Genetics, Ghent University, University Hospital Ghent, Medical Research Building 1, De Pintelaan 185, B-9000 Ghent, Belgium; 3 Division of Biology & Biological Engineering, California Institute of Technology, MC156-29, 1200 East California Boulevard, Pasadena, California 91125, USA

## Abstract

The gradual reprogramming of haematopoietic precursors into the T-cell fate is characterized by at least two sequential developmental stages. Following Notch1-dependent T-cell lineage specification during which the first T-cell lineage genes are expressed and myeloid and dendritic cell potential is lost, T-cell specific transcription factors subsequently induce T-cell commitment by repressing residual natural killer (NK)-cell potential. How these processes are regulated in human is poorly understood, especially since efficient T-cell lineage commitment requires a reduction in Notch signalling activity following T-cell specification. Here, we show that GATA3, in contrast to TCF1, controls human T-cell lineage commitment through direct regulation of three distinct processes: repression of NK-cell fate, upregulation of T-cell lineage genes to promote further differentiation and restraint of Notch activity. Repression of the Notch1 target gene *DTX1* hereby is essential to prevent NK-cell differentiation. Thus, GATA3-mediated positive and negative feedback mechanisms control human T-cell lineage commitment.

## Abstract (web summary)

Strong Notch signalling promotes initial T cell lineage specification of lymphoid progenitors but is also permissive for thymic natural killer (NK) cell development. Here the authors show that GATA3 directs human T-lineage commitment by modulating Notch activity and repressing the NK programme.

## Graphical abstract

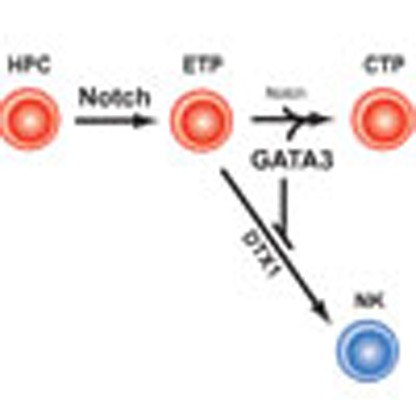

T cell development is a tightly regulated process in which multipotent haematopoietic precursor cells (HPCs) are gradually converted into committed T-cell progenitors^1^,^2^. This is orchestrated by a complex network of molecular regulators, each contributing to several stages of early T cell development^3^,^4^. Studies in mice revealed that T cell development is initiated in thymus colonizing multipotent HPCs through Notch signalling activity that induces T-lineage specification^5^,^6^,^7^. This is associated with T cell factor (TCF)1-dependent induction of T cell specific genes^8^,^9^, as well as GATA3-mediated repression of B-lineage potential^10^,^11^. Nevertheless, other developmental options, such as NK-cell potential, are still retained within these cells. Subsequently, commitment into the T cell pathway is induced through a Bcl11B-dependent mechanism that actively represses NK cell development^12^,^13^,^14^.

In human, similar developmental stages of early T cell development exist, but the molecular processes that control them are less clear. While the requirement for strong NOTCH1 signalling to induce T-lineage specification is well-established^15^,^16^, studies from our lab and others have revealed some remarkable differences in how this pathway controls later stages of T cell development in human compared to in mouse, with strong Notch-dependent TCR-γδ development in human as the most remarkable difference^15^,^17^,^18^,^19^. However, these studies also revealed that Notch signalling is permissive for NK cell development^20^, indicating that Notch activation is not sufficient to induce T-cell commitment, in agreement with other studies^7^,^21^. Moreover, following the strong NOTCH1-dependent T-lineage specification step, induction of human T-lineage commitment and further differentiation into αβ-lineage double positive (DP) thymocytes occurs more efficiently when Notch signalling activity is reduced^15^,^22^. In agreement, Notch target genes that require the highest level of Notch activation (such as *NRARP* and *DTX1*) are repressed during the commitment process, while others (such as *HES1* and *MYC*) remain expressed until the cells have passed through the β-selection checkpoint^1^,^2^,^15^,^22^. While changes in Notch signal strength could explain such differential regulation, other transcriptional regulators may also control the expression of Notch target genes, as previously illustrated for E-protein dependent *HES1* expression^23^. Indeed, when the expression patterns of known Notch target genes are studied individually, it is clear that other regulatory inputs are required to explain the diversity in expression^2^,^15^, a phenomenon that is also observed during mouse T cell development^24^.

Given that Notch signalling is not sufficient to control human T-lineage commitment, we investigated which other transcription factors mediate this process. We focused on TCF1 and GATA3, two essential regulatory proteins during T cell development, and show that GATA3, but not TCF1, controls the human T-lineage commitment process. We demonstrate that TCF1 requires Notch activation to induce T-lineage specification, whereas GATA3 is required to induce T-lineage commitment through direct regulatory roles that lead to repression of NK-cell fate and progression along the T developmental pathway. In addition, GATA3 provides a negative feedback onto the Notch signalling pathway in which repression of *DTX1* is required to prevent diversion into the NK-cell pathway. Overall, our work reveals that GATA3 is required to shut off NK-cell development and to restrict Notch signalling activity to promote T-cell commitment in human T-cell progenitors.

## Results

### Notch signalling is insufficient to induce T-cell commitment

**Figure 1:**
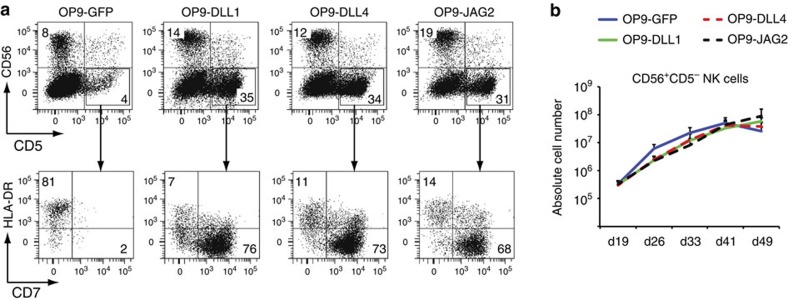
Notch signalling is permissive for human NK cell development. (**a**) Flow cytometry analysis of CB CD34^+^lin^−^ precursors after 18 days of coculture on OP9 stromal cells expressing different Notch ligands, as indicated above the dot plots, and in the presence of IL7, SCF, FLT3L and IL15. Dot plots show analysis of CD56 versus CD5 staining (upper plots) and HLA-DR versus CD7 staining (lower plots, gated on CD5^+^CD56^−^ cells). NK-lineage cells are identified as CD56^+^CD5^−^ and T-lineage cells as CD5^+^CD7^+^CD56^−^HLA-DR^−^. (**b**) Graphs show the kinetics of the CD56^+^CD5^−^ NK cell numbers generated on OP9 stromal cells expressing different Notch ligands at indicated time points. Data shows average of three independent experiments and error bars indicate s.e.m.

Notch signalling is essential to induce T-lineage specification in both mouse and human but its role in human T-cell commitment is less clear. We previously documented that human T-cell precursors efficiently develop into NK cells when Notch signalling is activated through intracellular Notch (ICN)1 overexpression or OP9-Delta-like ligand 1 (DLL1) exposure^20^, and that developmental progression into DP thymocytes is more efficient when Notch activity is reduced^15^. Since we now demonstrated that human thymic epithelial cells mainly express the Notch ligands Delta-Like-4 and Jagged2 (ref. 25), we re-evaluated our previous work by studying the impact of both Notch ligands on human NK cell development in comparison to DLL1. Both CD56^+^CD5^−^ NK-lineage and CD56^−^CD5^+^CD7^+^HLA-DR^−^ T-lineage cells (called CD5^+^CD7^+^ hereafter) were generated from cord blood (CB) derived HPCs on OP9 stromal cells that express either DLL1, DLL4 or JAG2 (Fig. 1a), and NK cell numbers (Fig. 1b) from these cultures were comparable with OP9-green fluorescent protein (GFP) cultures in which no T-lineage supporting Notch activation occurs as evident from the lack of CD5^+^CD7^+^ cells (Fig. 1a). Thus, Notch signalling by itself is not sufficient to discriminate between NK- and T-cell lineages.

### TCF1 requires notch to induce human T-lineage specification

**Figure 2:**
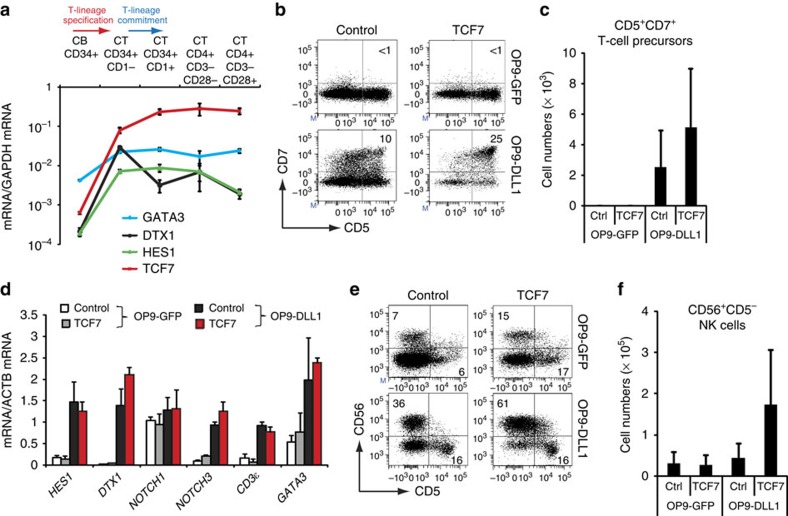
TCF1 requires Notch activation to induce T-lineage specification. (**a**) Quantitative PCR of *GATA3*, *DTX1*, *HES1* and *TCF7* expression during early human T cell development in different cell populations as indicated. Data shows the average expression in 3–4 independent samples, and error bars indicate s.e.m. (**b**) Flow cytometry analysis of control and *TCF7*-transduced CB CD34^+^lin^−^ precursors in OP9-GFP or OP9-DLL1 cocultures in the presence of IL7, SCF and FLT3L, showing the development of CD5^+^CD7^+^ early T cell precursors after 6 days of coculture. (**c**) Absolute numbers of CD5^+^CD7^+^ T precursor cells developed in corresponding cultures from **b**. Data shows average of three independent experiments and error bars indicate s.e.m. (**d**) Quantitative real-time RT-PCR gene expression analysis of Notch and T cell-related genes in control (white and black bars) or *TCF7* (grey and red bars) transduced human CB CD34^+^ after 2 days of coculture on OP9-GFP (white and grey bars) or OP9-DLL1 (black and red bars), relative to *ACTB* levels and relative to one control-transduced sample cultured on OP9-DLL1. Data shows average expression in two independent samples. Error bars indicate s.e.m. (**e**) Flow cytometry analysis of control or *TCF7*-transduced CD34^+^lin^−^ precursors cells in the presence of IL7, SCF, FLT3L and IL15 on OP9-GFP or OP9-DLL1 stromal cells after 13 days of coculture. (**f**) Corresponding absolute number of CD56^+^CD5^−^ NK cells in cocultures from **e**. Data shows average of three independent experiments and error bars indicate s.e.m.

In addition to Notch signalling, both TCF1 and GATA3 are required during early T cell development and gene expression analysis in *ex vivo* isolated T-cell precursors shows a strong induction of Notch signalling (*HES1* and *DTX1* expression) and *TCF7* (coding for TCF1) expression in human child thymus-derived intrathymic CD34^+^CD1^−^ T-lineage specified precursors (Fig. 2a) compared with extrathymic CB CD34^+^ precursors (Fig. 2a). Baseline *GATA3* expression in these input multipotent CB CD34^+^ HPCs is higher as compared with *HES1*, *DTX1* and *TCF7*, and shows a more subtle increase towards CD34^+^CD1^−^ T-cell precursors (Fig. 2a).

Since TCF1 is sufficient in the absence of Notch activation to initiate the T-lineage programme in mice^8^, we investigated if TCF1 alone or TCF1 and Notch activity combined could induce T-lineage commitment at the expense of NK cell differentiation in human HPCs. Strikingly, enforced *TCF7* expression in human CD34^+^lin^−^ CB cells was not sufficient to support the generation of early CD34^+^CD7^+^ (Supplementary Fig. 1a,b) and CD5^+^CD7^+^ T-lineage progenitors in the absence of Notch signalling (Fig. 2b), despite obtaining similar expression levels in transduced cells compared with *in vivo* thymocytes (Supplementary Fig. 1c). In conjunction with Notch signalling, *TCF7* slightly, but not significantly, enhanced differentiation towards CD5^+^CD7^+^ T-cell precursors (Fig. 2b,c), and gene expression analysis revealed a strict requirement for Notch signalling to induce Notch target genes as well as T-cell genes such as *CD3ɛ* and *GATA3* (Fig. 2d). In NK-cell permissive cultures, enforced *TCF7* expression did not repress NK cell development on OP9-GFP stromal cells and even enhanced their development when Notch signalling was present (Fig. 2e,f). These findings indicate that TCF1 requires Notch activation to induce human T-lineage specification, in contrast to in mice^8^, and that TCF1, like Notch, is not sufficient to discriminate between NK- and T-cell lineages.

### High-level GATA3 inhibits notch-induced T-cell specification

**Figure 3:**
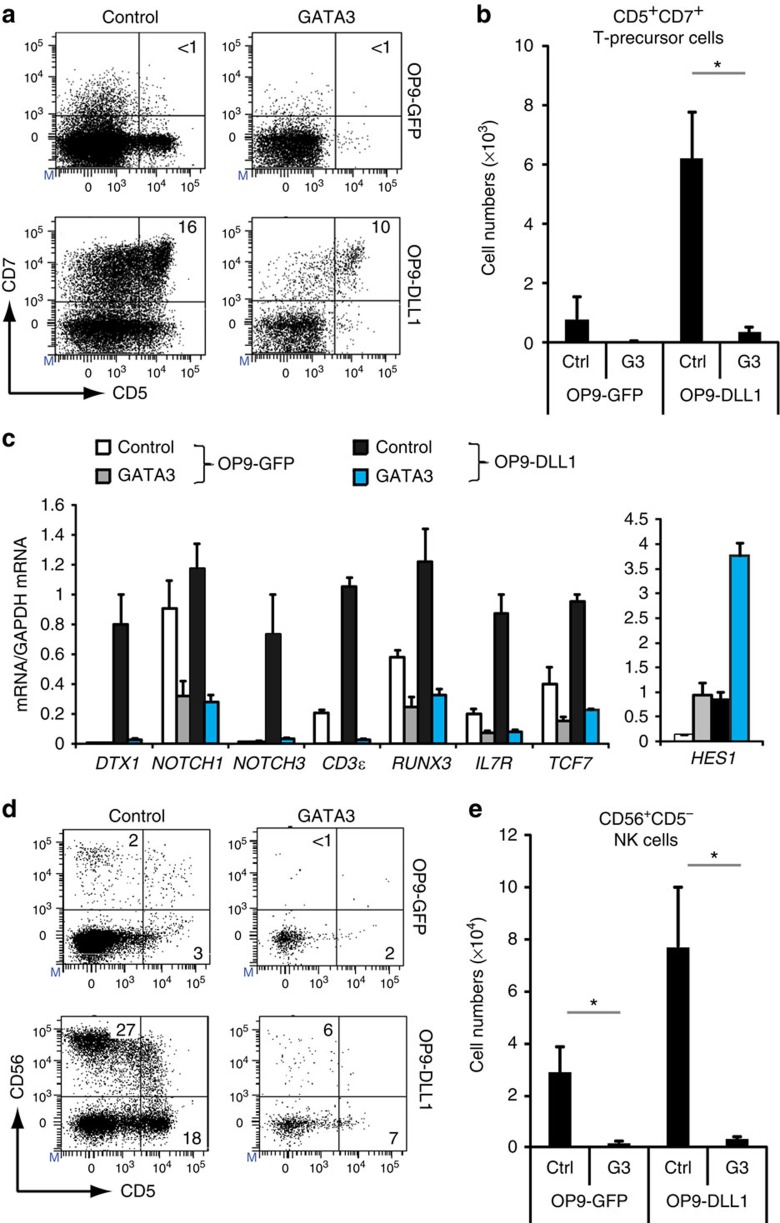
High-level GATA3 expression inhibits Notch-induced T-lineage specification. (**a**) Flow cytometry analysis of control and *GATA3*-transduced CB CD34^+^lin^−^ precursor cells in OP9-GFP and OP9-DLL1 cocultures in the presence of IL7, SCF and FLT3L, showing the development of CD5^+^CD7^+^ T precursor cells after 6 days of coculture. (**b**) Graphs show absolute number of CD5^+^CD7^+^ cells generated in corresponding cultures shown in **a**. Data shows the average of seven independent experiments and error bars indicate s.e.m. *P<0.05 (non-parametric paired Wilcoxon test) (**c**) Quantitative PCR analysis of changes in gene expression in control (white and black bars) and *GATA3* (grey and blue bars) transduced CB CD34^+^lin^−^ precursors after 2 day coculture on OP9-GFP (white and grey bars) and OP9-DLL1 (black and blue bars). Data shows the average expression of two independent experiments, relative to *GAPDH* levels and relative to one control-transduced sample cultured on OP9-DLL1. Error bars indicate s.e.m. (**d**) Flow cytometry analysis of control and *GATA3*-transduced CB CD34^+^lin^−^ precursor cells in OP9-GFP or OP9-DLL1 cocultures in the presence of IL7, SCF, FLT3L and IL15, showing the development of CD56^+^ NK cells or CD5^+^ T precursor cells after 13 days of coculture. (**e**) Graphs show absolute numbers of CD56^+^CD5^−^ NK cells generated in corresponding cultures shown in **d**. Data shows the average of seven independent experiments and error bars indicate s.e.m. *P<0.05 (non-parametric paired Wilcoxon test).

We subsequently investigated whether GATA3 was capable of inducing human T-lineage commitment at the expense of natural killer (NK) cell development. Exogenous GATA3 raised overall *GATA3* messenger RNA (mRNA) expression in CB CD34^+^ HPCs (Supplementary Fig. 2a) to a similar relative level as for TCF7 following transduction (Supplementary Fig. 1c), reaching around 10-fold higher levels compared with *in vivo* DP thymocytes (Supplementary Fig. 2a). Like TCF1, GATA3 was unable to replace the Notch requirement to induce T-lineage specification as no CD34^+^CD7^+^ (Supplementary Fig. 2b,c) and CD5^+^CD7^+^ T-cell precursors developed in OP9-GFP cultured *GATA3* transduced cells (Fig. 3a), in agreement with observations in mice^26^,^27^. In contrast to *TCF7*, *GATA3* expression also impaired the generation of CD34^+^CD7^+^ (Supplementary Fig. 2b,c) and CD5^+^CD7^+^ early T cell precursors in the presence of Notch signalling (Fig. 3a,b), suggesting that elevated GATA3 levels may interfere with Notch activation. This was revealed through gene expression analysis after 48 h short-term exposure to DLL1 to discriminate direct regulatory effects by GATA3 from altered gene expression that results from changes in cellular composition (Fig. 3c). GATA3 repressed induction of the Notch1 target genes *DTX1*, *NOTCH3*, *CD3E*, *RUNX3*, *IL7R* and *TCF7*, and reduced expression of *NOTCH1* itself (Fig. 3c). *HES1* expression was upregulated by GATA3, even without Notch activation, consistent with observations in mice^27^. Unlike *TCF7*, elevated *GATA3* expression inhibited CD56^+^ NK cell development on both OP9-control and OP9-DLL1 (Fig. 3d,e). Thus, whereas TCF1 synergizes with Notch activation to induce T-lineage specification and supports both T and NK cell development, simultaneous GATA3 and Notch activation in HPCs results in inhibition of T-lineage specification due to GATA3-mediated interference of Notch activation.

### GATA3-mediated restraint of notch concurs with T commitment

**Figure 4:**
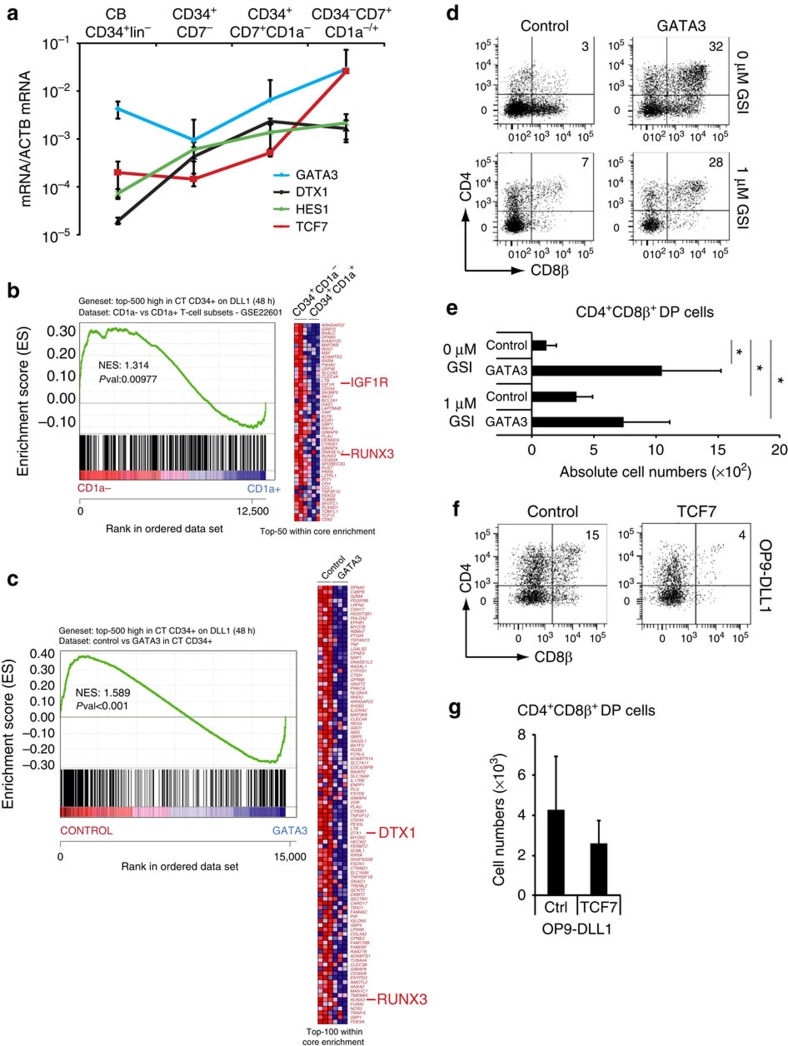
GATA3 restrains Notch activation at the T-lineage commitment stage. (**a**) Quantitative PCR of *GATA3*, *DTX1*, *HES1* and *TCF7* expression in different stages of *in vitro* generated T cell precursors from CB CD34^+^lin^−^ HPCs after 7 days of OP9-DLL4 coculture. Data shows the average expression in 3–4 independent samples on a log scale and erros bars indicate s.e.m. (**b**,**c**) GSEA shows a significant enrichment of the top 500 Notch-dependent genes in human CD34^+^ thymocytes in the set of genes higher expressed in (**b**) uncommitted CD34^+^CD1a^−^ versus CD34^+^CD1a^+^ committed T-cell precursors^50^, and genes expressed higher in (**c**) control versus GATA3-transduced CD34^+^ thymocytes as determined by microarray after 48 h of transduction. (**d**) Flow cytometry analysis of control and *GATA3*-transduced CD34^+^CD1^−^ uncommitted thymocytes in OP9-DLL1 cocultures with addition of 0 or 1 μM GSI and in the presence of IL7, SCF and FLT3L, showing the development of CD4^+^CD8β^+^ DP thymocytes after 6 days of coculture. (**e**) Graph show absolute number of CD4^+^CD8β^+^ DP thymocytes, generated in corresponding cultures shown in **d**. Data shows the average of four independent experiments and errors bars show s.e.m. *P<0.05 (non-parametric paired Wilcoxon test) (**f**) Flow cytometry analysis of control and *TCF1* transduced CD34^+^CD1^−^ uncommitted thymocytes in OP9-DLL1 cocultures in the presence of IL7, SCF and FLT3L, showing the development of CD4^+^CD8β^+^ DP thymocytes after 19 days of coculture. (**g**) Graph shows absolute number of CD4^+^CD8β^+^ DP thymocytes, generated in corresponding cultures shown in **a**.

While our *ex vivo* isolated progenitor subsets revealed simultaneous induction of *TCF7*, *GATA3* and Notch target gene expression (Fig. 2a) within the most immature human CD34^+^CD1a^−^ thymocytes, the heterogeneity of this population may obscure the detection of discrete developmental stages of early T cell development that could reveal kinetic differences in the expression of these genes. Therefore, we re-evaluated induction of *GATA3* and *TCF7* expression following Notch-induced T cell development using *in vitro* generated stages of human T cell development, as previously used^28^. This revealed that the induction of *TCF7* and *GATA3* is delayed compared with the direct Notch targets *DTX1* and *HES1*, when sequential stages of T cell development are evaluated (Fig. 4a), consistent with earlier findings in both mouse and human^7^,^21^. Thus, instead of acting simultaneously, TCF1 and GATA3 may act sequentially following initial Notch signalling to induce T cell commitment at the expense of NK cell differentiation.

Since earlier work from our lab revealed that a reduction in Notch signalling activity enhances human T-lineage commitment and differentiation into DP thymocytes, we focused on GATA3 because its negative effect on Notch activation, as characterized above, could provide a molecular mechanism that mediates this process. Indeed, gene set enrichment analysis (GSEA) revealed that the most significant Notch-dependent genes in human CD34^+^ thymocytes, including *IGFR1* and *RUNX3* (ref. 29), are downregulated during the CD34^+^CD1a^−^ to CD34^+^CD1a^+^ transition that marks human T-lineage commitment (Fig. 4b), confirming an initial but partial reduction in Notch signalling activity at this developmental stage as also previously illustrated for the Notch targets *DTX1* and *NRARP* (ref. 15). Furthermore, GATA3 transduction results in repression of these Notch-dependent genes in human CD34^+^ thymocytes (48 h after transduction, Fig. 4c, Supplementary Data 1), suggesting a negative feedback of GATA3 onto the Notch pathway during the induction of T-lineage commitment.

To functionally test this hypothesis, we introduced GATA3 into CD34^+^CD1a^−^ T-lineage specified thymocytes, cells that already received strong Notch signalling *in vivo*. In this setting, *GATA3* transduction also resulted in 10-fold higher *GATA3* levels compared with in *in vivo* thymocytes (Supplementary Fig. 3), thus yielding similar levels as in CB transduction experiments (Supplementary Fig. 2a). Consistent with previous experiments^15^, reduction of Notch signalling activity by adding a low, 1 μM concentration of the γ-secretase inhibitor (GSI) 7 (*N*-[*N*-(3,5-difluorophenyl)-l-alanyl]-s-phenyl-glycine t-butyl ester) (DAPT) in control-transduced cells resulted in an accelerated development towards the DP stage, in comparison to when Notch signalling was present (0 μM GSI, Fig. 4d). Interestingly, elevated *GATA3* levels in the presence of Notch signalling also resulted in such an enhanced differentiation towards the DP stage (Fig. 4d) and yielded higher numbers of DP thymocytes at that time (Fig. 4e). In contrast, *TCF7* expression did not accelerate T cell development (Fig. 4f,g). Thus, following Notch induced T-lineage specification, GATA3 can restrain Notch signalling activity to promote further T-lineage differentiation, illustrating context-specific effects of GATA3 on human T cell development.

### GATA3 is essential to prevent NK cell development

**Figure 5:**
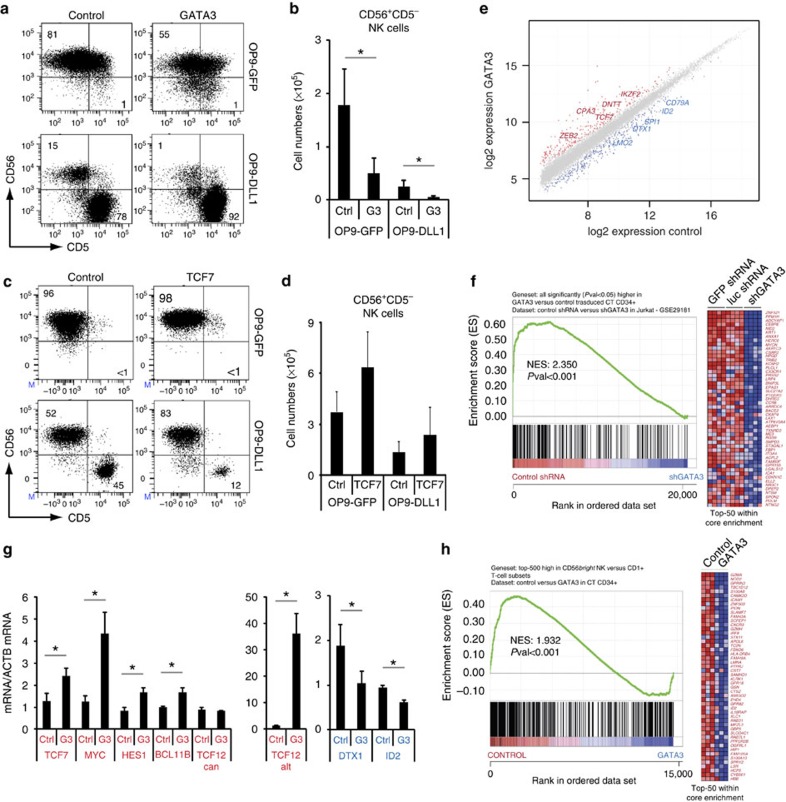
GATA3 inhibits NK cell development. (**a**) Flow cytometric analysis of control and *GATA3*-transduced CD34^+^CD1^−^ uncommitted thymocytes in OP9-GFP or OP9-DLL1 cocultures in the presence of IL7, SCF, FLT3L and IL15, showing the development of CD56^+^ NK cells or CD5^+^ T precursor cells after 13 days of coculture. Absolute numbers of CD56^+^CD5^−^ NK cells are depicted in **b**. Data shows the average of seven independent experiments and errors bars show s.e.m. *P<0.05 (non-parametric paired Wilcoxon test) (**c**) Flow cytometry analysis of control and *TCF7*-transduced CD34^+^CD1^−^ uncommitted thymocytes in OP9-GFP or OP9-DLL1 cocultures in the presence of IL7, SCF, FLT3L and IL15, showing the development of CD56^+^ NK cells or CD5^+^ T precursor cells after 13 days of coculture. Absolute numbers of CD56^+^CD5^−^ NK cells are depicted in **d**. (**e**) Double log scatter plot showing genes with significant differential expression between GATA3 and control-transduced CD34^+^ thymocytes. Data shows significant differential expressed genes over three independent experiments. Red and blue dots represent the significant differentially expressed genes (adjusted *P* value <0.05). (**f**) GSEA shows a significant enrichment of genes upregulated following GATA3 overexpression in thymic CD34^+^ progenitors in the set of genes higher expressed in control shRNA versus GATA3 shRNA transduced Jurkat cells^31^ (**g**) Quantitative PCR analysis of changes in gene expression in control and *GATA3*-transduced CD34^+^ thymocytes after 2 day coculture on OP9-DLL1. Data shows the average expression of two independent experiments, relative to *ACTB* levels and relative to one control-transduced sample cultured on OP9-DLL1. Error bars indicate s.e.m. *P<0.05 (paired Student's *t*-test) (**h**) GSEA shows a significant enrichment for CD56^bright^ human NK cells signature genes in control versus GATA3-transduced human CD34^+^ thymocytes.

Human T-lineage commitment is not only characterized by a functional requirement to reduce Notch activation, but also demands loss of NK-cell potential. In agreement with a regulatory role to induce T-cell commitment, GATA3 repressed the development of CD56^+^CD5^−^ NK cells in Notch activated T-lineage specified precursors compared with the control (Fig. 5a,b). In contrast, *TCF7* transduction did not reduce NK cell development from intrathymic T-cell precursors (Fig. 5c,d). To investigate GATA3-induced molecular changes, we performed microarray-based gene expression profiling in 48-h control and GATA3-transduced CD34^+^ thymocytes (Supplementary Data 1). GATA3-induced significant upregulation of several T-lineage associated genes such as *TCF7*, *DNTT*, *IKZF2*, *GFI1B* (ref. 30) and *ZEB2* (Fig. 5e, Supplementary Data 1), and GSEA confirmed that GATA3 upregulated genes were functionally GATA3-dependent in published^31^ knockdown experiments (Fig. 5f). Although *BCL11B* did not reach significance in the microarrays, quantitative reverse transcription PCR confirmation experiments (Fig. 5g) did reveal significant *BCL11B* upregulation by GATA3, and intriguingly also *TCF12alt* (coding for HEBalt), not *TCF12*can, consistent with a role for HEBalt in inducing T-lineage commitment^32^. In contrast to Notch target genes that are downregulated during T-lineage commitment which are repressed by GATA3, such as *DTX1* (Fig. 2a and Figs 4c and 5e,g) and RUNX3 (Fig. 4b,c), GATA3-enhanced expression of *MYC* and *HES1* (Fig. 5g), Notch targets that remain expressed during the subsequent Notch-dependent stages of human T cell development, illustrating that GATA3 selectively restrains Notch targets. In addition, GATA3 significantly repressed ‘stem cell' genes whose downregulation is essential for further T cell development (*LMO2*, *MEF2C* and *SPI1*)^33^,^34^, and/or that are involved in the development of other haematopoietic lineages such as *CD79A* for B cell development (downregulation of *EBF1*, expressed at low levels, was observed but just failed to reach significance, *P*=0.052) and *ID2* for NK-lineage differentiation^35^ (Fig. 5e, Supplementary Data 1). Additional GSEA for specific cytotoxic CD56^dim^ (Supplementary Fig. 4) and cytokine-producing CD56^bright^ (Fig. 5h) human NK-cell signatures revealed selective loss of these gene sets, including *ID2* (not within the top-50 core-enriched genes, Fig. 5h), on GATA3 expression in both cases, confirming that GATA3 antagonizes the induction of NK-lineage genes in early T cell precursors. Unlike in murine studies^27^, GATA3 overexpression did not induce mast cell development in human uncommitted thymocytes (both in the presence or absence of Notch activation, Supplementary Fig. 5a), although we are able to generate FcERI^+^CD117^+^ mast cells from CD34^+^ CB HPCs (Supplementary Fig. 5b). While mast cell lineages genes were upregulated on GATA3 overexpression (Supplementary Fig. 5c), similar as in mice^27^, critical T cell genes also remained expressed that were repressed in the murine experiments, confirming maintenance of T-lineage identity in agreement with the enhanced differentiation into DP thymocytes (Fig. 4d,e).

**Figure 6:**
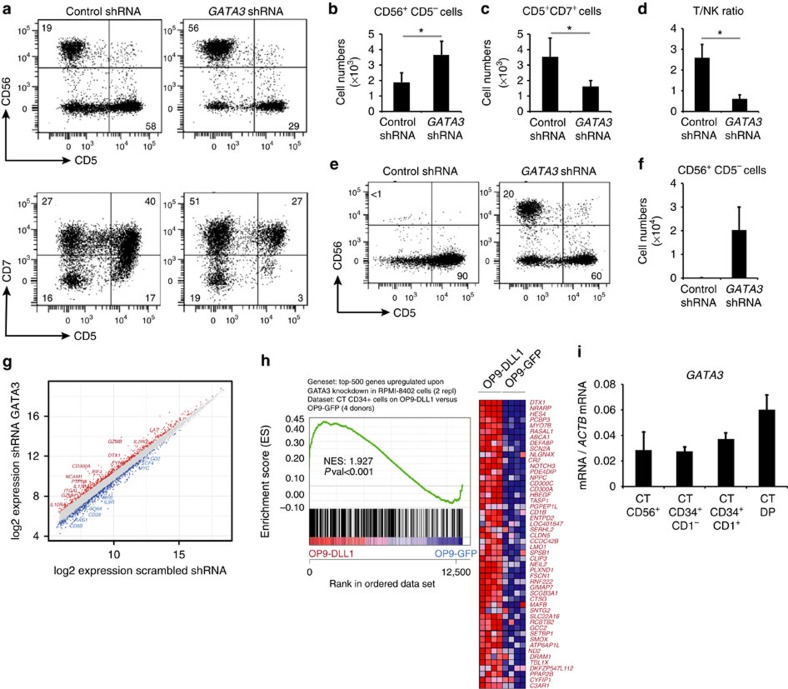
GATA3 is critical to initiate T-lineage commitment at the expense of NK cell differentiation. (**a**) Flow cytometry analysis of control shRNA and *GATA3* shRNA transduced CD34^+^ CB progenitors in 2-week OP9-DLL1 cocultures in the presence of IL7, SCF, FLT3L and IL15. (**b**) Number of CD56^+^CD5^−^ NK cells developed in corresponding cultures from **a**. (**c**) Number of CD5^+^CD7^+^ T-lineage precursors developed in corresponding cultures from **a**. (**d**) T/NK cell ratio from cultures depicted in **a**. Data shows the average of six independent experiments and error bars indicate s.e.m. *P<0.05 (non-parametric paired Wilcoxon test) (**e**) Flow cytometry analysis of control shRNA and *GATA3* shRNA transduced CD34^+^ CB progenitors in 2-week OP9-DLL1 cocultures in the presence of IL7, SCF and FLT3L. (**f**) Number of CD56^+^ NK cells developed in corresponding cultures from **e**. Data shows the average of four independent experiments and error bars indicate s.e.m. (**g**) Double log scatter plot showing genes with significant differential expression between shRNA GATA3 and shRNA control-transduced RPMI-8402 cells. Data shows significant differential expressed genes over two independent experiments. Red and blue dots represent the significant differentially expressed genes (adjusted *P* value<0.05). (**h**) GSEA shows a significant enrichment for genes that are upregulated following GATA3 shRNA-mediated knockdown in Notch-dependent genes in OP9-DLL1 versus OP9-GFP cocultured thymocytes (**i**) Quantitative PCR for *GATA3* expression in various cell subsets from thymus (CT): CD56^+^ NK cells, CD34^+^CD1^−^ uncommitted and CD34^+^CD1^+^ committed progenitors and DP thymocytes. Data shows average expression, relative to *ACTB*, of 4–6 independent samples and error bars indicate s.e.m.

To investigate whether GATA3 is essential to induce T-lineage commitment at the expense of NK cell development, we knocked-down GATA3 levels through GATA3 directed short hairpin RNAs (shRNAs; Supplementary Fig. 6a), resulting in a 60% knockdown in CD34^+^ thymocytes (Supplementary Fig. 6b). GATA3 reduction significantly increased both the frequency (Fig. 6a) and absolute number (Fig. 6b) of NK cells, while the number of CD7^+^CD5^+^ T cell precursors significantly decreased (Fig. 6c), thereby causing a fivefold reduction in the T/NK cell ratio compared with the control (Fig. 6d). Remarkably, in the absence of IL15 when virtually no NK cells develop in OP9-DLL1 cocultures (control shRNA, Fig. 6e,f), shRNA-mediated knockdown of GATA3-induced NK cell differentiation (Fig. 6e,f), indicating that GATA3 is required to inhibit NK cell development in Notch activated precursor cells. These differential effects of *GATA3* shRNA knockdown on T and NK cell development were also apparent following overexpression of repressor of GATA3 (ROG (ref. 36)), although the increase in NK cell numbers failed to reach significance (Supplementary Fig. 7a–c). Gene expression analysis following GATA3 shRNA-mediated knockdown revealed an increase of several NK cell associated genes compared with control shRNA transduced cells, while expression of T cell genes was reduced (Fig. 6g, Supplementary Data 2). Direct comparison of shRNA-mediated *GATA3* knockdown versus *TCF7* knockdown further illustrated the unique roles for both transcriptional regulators since NK cell genes (including *NFIL3, NCAM1, GZMB* and *GZMH*) were upregulated on *GATA3* knockdown compared with when *TCF7* expression was reduced (Supplementary Fig. 8, Supplementary Data 3). Also Notch-dependent genes were significantly upregulated (Fig. 6h), including canonical Notch target genes DTX1, NRARP, HES4 and NOTCH3, confirming that GATA3 represses Notch activation, in agreement with our GATA3 overexpression results. Consistent with the requirement for higher GATA3 levels to support T cell differentiation compared with NK-lineage development, *GATA3* mRNA levels rise on differentiation of CD34^+^CD1^−^ uncommitted T cell precursors into DP thymocytes, whereas intrathymic CD56^+^ NK cells maintain similar GATA3 levels as uncommitted T cell precursors (Fig. 6i). These results therefore illustrate that an increase in GATA3 expression is essential to induce T-lineage commitment at the expense of NK cell development.

### Direct regulatory roles of GATA3 during T-cell commitment

**Figure 7:**
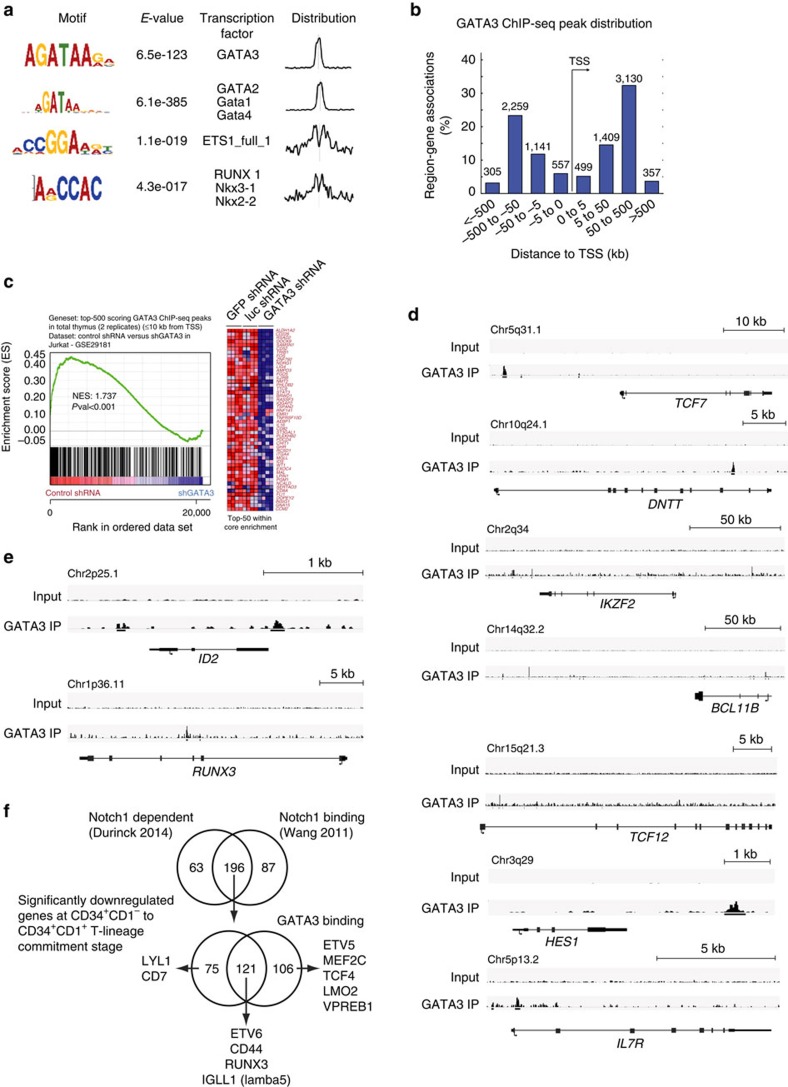
Direct regulatory roles for GATA3 during induction of T-lineage commitment. (**a**) GATA3 binding motif analysis following GATA3 ChIP-sequencing (ChIP-Seq) in total human thymocytes. (**b**) Distribution of GATA3 peaks around the transcriptional start site (TSS) of protein coding genes. (**c**) GSEA shows significant enrichment of the top 500 ChIP-Seq peaks in the gene set that is significantly higher expressed in control shRNA versus GATA3 shRNA transduced Jurkat cells showing high correlation between GATA3 binding and GATA3-dependent regulation. (**d**) GATA3 binding at selected gene loci that are associated with T cell development. (**e**) GATA3 binding at selected gene loci that are associated with NK cell development. (**f**) Top Venn diagram shows overlap between Notch dependent and Notch1 bound loci for genes that show significant downregulation at the CD34^+^CD1a^−^ to CD34^+^CD1a^+^ T-lineage commitment stage. The Venn diagram at the bottom shows overlap between Notch-regulated and GATA-3 regulated genes (bottom).

To reveal direct regulatory roles for GATA3 during human T-lineage commitment, we performed chromatin immunoprecipitation (ChIP)-sequencing experiments in human postnatal thymocytes to study genome-wide patterns of GATA-3 binding. Peaks were enriched for GATA binding motifs at the centre, with frequent neighbouring sites for RUNX1 and ETS1 (Fig. 7a). Binding predominantly occurred at sites >50 kb away from the transcriptional start site (Fig. 7b) and most bound regions displayed functional GATA3 dependency^31^ (Fig. 7c). GATA3 binding was observed at several critical T-lineage specific genes (*TCF7*, *DNTT*, *IKZF2*, *BCL11B* and *TCF12*, Fig. 7d) and at the Notch target *HES1*, genes that were upregulated following *GATA3* overexpression (Fig. 5e,g), consistent with data from mouse^28^, identifying sites through which GATA3 can contribute to the expression of several key genes required for further T cell development. GATA3 binding was also observed at the *IL7R* promoter (Fig. 7d), a feature that seems unique for primates as the corresponding GATA site is conserved in chimp and gorilla (Supplementary Fig. 9a), but not in rodents^28^ (Supplementary Fig. 9a,b).

Consistent with the functional ability of GATA3 to repress NK cell development, GATA3 binding was observed in 30% of the repressed NK-signature genes (including *ID2* and *RUNX3*, Fig. 7e, Supplementary Data 4 and 5), suggesting both direct and indirect repressive mechanisms of GATA3 on NK cell development. Also, around half of the top 500 genes from the NK cell signature gene set displayed functional Notch dependency and one third of those show Notch1 binding (Supplementary Data 6)^37^, further correlating Notch activation with NK cell development. Direct comparison of GATA3 binding in human versus mouse revealed some differences in GATA3 occupancy at important genes involved in these processes, including *CD300A* (coding for the NK inhibitory receptor IRp60), *ID2*, *IL7RA* and *MYC* (Supplementary Fig. 9b).

A high degree of Notch- and GATA3-dependent regulation was observed for the 425 genes that were significantly downregulated at the CD34^+^CD1^−^ to CD34^+^CD1^+^ stage that marks human T-lineage commitment (Supplementary Data 7). Of these, 259 genes were shown to be functionally Notch dependent and the majority (196) shows Notch1 binding (Fig. 7f) in CUTTL1 cells^37^. Of these Notch-dependent genes, 121 also display GATA3 binding in human thymocytes, including genes such as *RUNX3*, *IGLL1* (coding for lamba5) and *ETV6*, suggesting a high degree of direct negative feedback regulation by GATA3 on early Notch-dependent genes (Fig. 7f). Some genes that were downregulated only reveal marks of GATA3-dependent regulation, such as *MEF2C*, *ETV5*, *VPREB*, *LMO2* and *TCF4*, others only show Notch1-dependent expression in CD34^+^CD1^−^ cells, including *CD7* and *LYL1* (Fig. 7f).

Overall, these data show that GATA3 directly controls progression of T-lineage differentiation, repression of NK cell differentiation and downmodulation of the Notch signalling pathway. Further GATA3 repressed genes also include stem cell and B-lineage genes, further supporting a critical role for GATA3 as a T-lineage driver.

### DTX1 is critical for thymus-derived NK cell development

**Figure 8:**
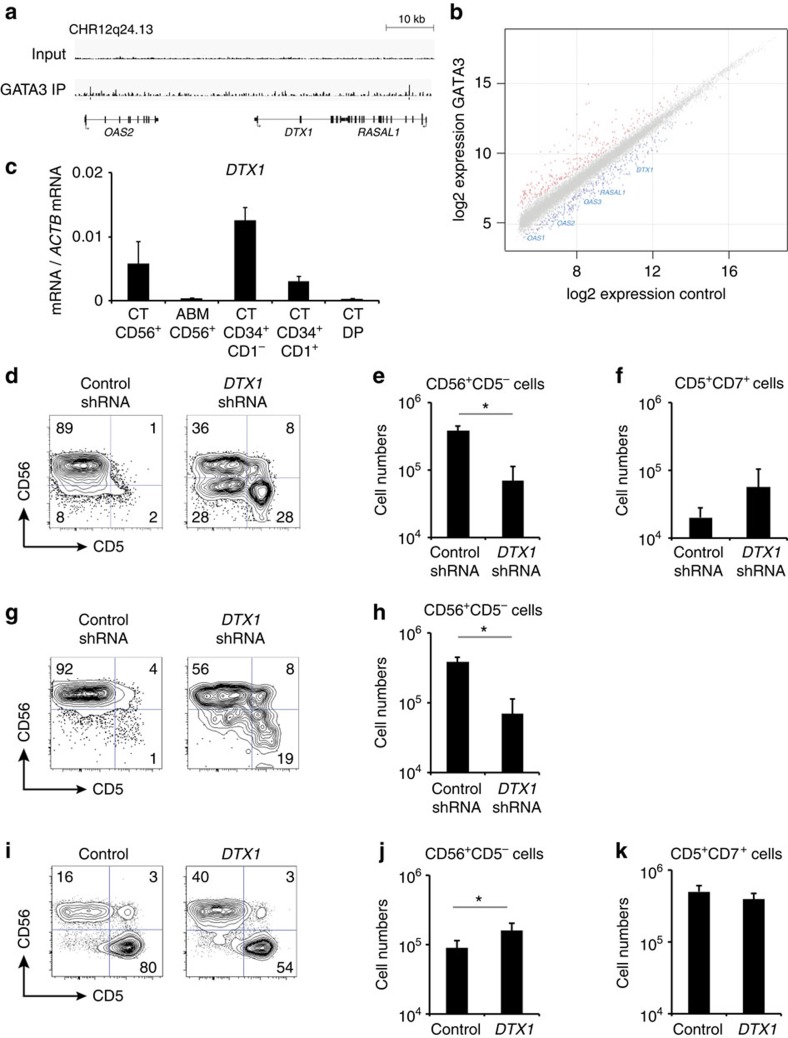
DTX1 is critical for thymus-derived NK cell development. (**a**) GATA3 binding at the DTX1 locus and the neighbouring RASAL1 and OAS2 loci. (**b**) Double log scatter plot showing similar GATA3-mediated repression of genes at the DTX1 locus in GATA3 versus control-transduced CD34^+^ thymocytes. Data shows the average of three independent experiments. Red dots represent the significant differentially expressed genes (adjusted *P* value <0.05). (**c**) Quantitative PCR for *DTX1* expression in thymus (CT) and adult bone marrow (ABM) CD56^+^ NK cells, compared with thymus-derived CD34^+^CD1^−^ uncommitted and CD34^+^CD1^+^ committed progenitors and DP thymocytes. Data shows average expression, relative to *ACTB*, of 3–6 independent samples and error bars indicate s.e.m. (**d**) Flow cytometry analysis of control shRNA and *DTX1* shRNA transduced CD34^+^lin^−^ CB progenitors in 2-week OP9-DLL1 cocultures in the presence of IL7, SCF, FLT3L and IL15. Number of NK cells (**e**) and T cells (**f**) developed in corresponding cultures from **d**. Data shows the average of four independent experiments with two different *DTX1* shRNAs and error bars indicate s.e.m. *P<0.05 (non-parametric paired Wilcoxon test) (**g**) Flow cytometry analysis of control shRNA and *DTX1* shRNA transduced CD34^+^CD1^−^ uncommitted thymocytes in 2-week OP9-GFP co-cultures in the presence of IL7, SCF, FLT3L and IL15. (**h**) Number of NK cells developed in corresponding cultures from **g**. Data shows the average of twp independent experiments with two different *DTX1* shRNAs and error bars indicate s.e.m. *P<0.05 (paired *t*-test) (**i**) Flow cytometry analysis of control and *DTX1* transduced CD34^+^CD1^−^ uncommitted thymocytes in 2-week OP9-DLL1 co-cultures in the presence of IL7, SCF, FLT3L and IL15. Number of NK cells (**j**) and T cells (**k**) developed in corresponding cultures from **i**. Data shows the average of five independent experiments and error bars indicate s.e.m. *P<0.05 (non-parametric paired Wilcoxon test).

The observations that Notch signalling supports human NK cell development and that induction of T-lineage commitment is associated with a reduction in Notch signalling activity and loss of NK cell potential suggests a link between both processes. We further focused on DTX1 since this is considered a negative regulator of Notch activity. Although GATA3 binding was not observed at the *DTX1* locus itself, peaks were detected in the flanking loci *RASAL1* and *OAS2* (Fig. 8a), genes that show similar GATA3-dependent repression in CD34^+^ thymocytes (Fig. 8b), suggesting that their expression is controlled through shared regulatory mechanisms. Intriguingly, compared with Notch-triggered CD34^+^CD1^−^ uncommitted thymocytes, *DTX1* expression levels remained higher in thymic NK cells compared with more differentiated thymocytes (Fig. 8c). This was not a simple reflection of Notch signalling activity due to the thymic microenvironment as illustrated by the lack of *HES1* expression in thymic NK cells (Supplementary Fig. 10). Virtually no *DTX1* expression was observed in adult bone marrow derived NK cells (Fig. 8c).

To investigate if *DTX1* is required for human NK cell development following Notch-induced T-lineage specification, we performed knockdown of *DTX1* expression using shRNA transduction in CB CD34^+^ HPCs in the presence of Notch signalling (OP9-DLL1). Compared with the control, NK cell development was significantly reduced on *DTX1* knockdown (Fig. 8d,e) and a small increase in the differentiation of CD5^+^CD7^+^ T-lineage cells was observed that however failed to reach significance (Fig. 8f). Intriguingly, in uncommitted CD34^+^CD1^−^ thymocytes in the absence of Notch activation (on OP9-GFP), knockdown of *DTX1* severely reduced NK cell development from T-lineage specified thymocytes (Fig. 8g,h), indicating that DTX1 is needed for thymus-derived NK cell development following Notch activation. Such a functional requirement is consistent with the *DTX1* expression that is observed in thymus NK cells (Fig. 8c). Conversely, *DTX1* overexpression resulted in a significant increase in NK cell development (Fig. 8i,j) and a small but non-significant reduction in T cell numbers was observed (Fig. 8k). Other overexpression experiments further confirmed that GATA3 and DTX1 act antagonistic during the human T/NK cell lineage choice (Supplementary Fig. 11a,b,c).

Thus, these findings reveal that DTX1 is essential for thymic NK cell development, highlighting the importance of GATA3 to repress *DTX1* expression to inhibit NK cell development.

## Discussion

Notch signalling is required to induce T cell development, but studies in both mouse and human have illustrated that activation of this pathway by itself is insufficient to impose T-cell fate. Here, we use functional gain- and loss-of-function experiments in primary human thymocytes, combined with genome-wide molecular data, to show that following Notch-induced human T-cell specification, GATA3, locks down T-lineage commitment by acting at three different levels. First, GATA3 prevents NK cell development by repressing NK-lineage associated genes. Second, GATA3 controls, both positive and negative, expression of Notch target genes and selectively represses those that are downregulated during human T-lineage commitment as exemplified by the Notch target gene *DTX1*. Third, GATA3 promotes T-lineage differentiation through upregulation of essential genes for T-cell development. Thus, GATA3 induces human T-lineage commitment through both positive and negative regulatory mechanisms following Notch and TCF1 mediated T-cell specification (Supplementary Fig. 12).

The positive regulatory role for GATA3 on the expression of other important transcription factors that mediate early T cell development, including *TCF7* and *BCL11B*, is consistent with observations in mice and illustrates that key components of the network are shared between species^1^,^3^,^4^. However, in mice it has been difficult to functionally reveal these positive roles of GATA3 using gain-of-function approaches as uncommitted thymocytes do not tolerate high levels of GATA3 in the presence of Notch signalling or are diverted to mast cells in its absence^27^,^34^. Here, we show that GATA3 can promote developmental progression of uncommitted human T cell precursors in the presence or absence of Notch signalling, consistent with earlier findings^38^. Thus, although human cells might be more tolerant to high levels of GATA3 compared with murine thymocytes, we propose that its interaction with other transcriptional programmes explains this difference in developmental outcome.

Indeed, previous studies from our lab have clearly illustrated differences in Notch activation status during early T cell development in human compared with in mice. While Notch activation in human peaks at T-cell specification and declines thereafter, Notch signalling continues to rise during murine T cell development until the β-selection checkpoint has been reached. Consistently, uncommitted early T cell precursors in human differentiate more efficiently into αβ-lineage DP thymocytes when Notch activity is reduced^15^,^19^, while murine ETPs remain strongly Notch-dependent until the DN3b β-selection stage to allow DP development^39^,^40^,^41^. As such, the negative feedback that GATA3 induces onto at least parts of the downstream Notch network will permit human cells to differentiate more efficiently, while murine thymocytes die as a result of the lack of Notch signalling activity during the early stages^39^. However, a similar GATA3-mediated mechanism may occur at β-selection during murine T cell development as positive effects of GATA3 become apparent at the later developmental stages^27^,^34^. In agreement, GATA3 repressive effects on *DTX1* have also been observed in mice^10^,^27^, indicating that similar mechanisms may be in play but at different stages of development. Therefore, we propose that GATA3 switches from a negative to a positive regulator of T cell development, as soon as the cells cross a Notch-dependent threshold. This would also explain why human uncommitted thymocytes are not diverted into mast cells, a property that is maintained in murine thymocytes until the peak of Notch activation is reached in DN3a thymocytes^27^.

Importantly, GATA3 does not repress all Notch target genes as illustrated by upregulation of *HES1* and *MYC*. This is important as Notch remains essential for the further development of T-lineage specified progenitor cells, although not at such high levels compared with earlier stages. This differential response of Notch target genes may be related to the individual role of each of the targets with respect to their effects on T cell development. Indeed, while it is clear that HES1 (ref. 42) and MYC (ref. 43) have positive roles during T-lineage development, DTX1 has been more considered as a negative regulator of Notch activity in mammals as illustrated through preferential B-lineage differentiation when overexpressed in mice^44^. Although the role of each of the GATA3 repressed Notch targets remains to be elucidated, GATA3 may be required to selectively downregulate some Notch targets that may be detrimental for further T cell development, thereby allowing the synergistic activity of Notch and GATA3 to drive further T cell differentiation^26^. The need for additional regulatory inputs to modulate different components of the Notch pathway has also been discussed during murine T cell development^24^, further supporting such distinct regulatory roles. It is tempting to speculate that such a GATA3 repressive mechanism onto the Notch pathway may be defective in early T-cell precursor acute lymphoblastic leukaemia (ETP-ALL) cases that are associated with GATA3 mutations^45^ as these may lead to aberrant Notch activation and inhibition of further T-lineage differentiation.

Although the inhibition of NK cell development by GATA3 may seem in conflict with observations in mice^46^, there are several aspects that differ in our experimental approach that may account for such differences. First, GATA3 levels differ between T and NK cells and, accordingly, the development of both lineages depends on such differential expression as evident from our gain- and loss-of-function approaches. Given that our shRNA reduced GATA3 expression ∼2-fold, our results are difficult to compare with full gene deletion approaches as used in murine experiments. Second, the absence of thymic NK cells in GATA3 deficient mice^46^ may reflect a defect in the generation of GATA3-dependent intrathymic T/NK cell precursors. Although human CD56^bright^ cells have been associated with GATA3-dependent intrathymic NK cells^46^, evidence now emerges that this NK cell subset represents a different stage of development compared with CD56^dim^ cells, rather than a separate NK-lineage with different origins^47^. We previously could not associate intrathymic NK cells with such a particular phenotype^20^ and thus the difference in GATA3 levels between both human subsets^46^ may not reflect a different origin. Given that we incompletely knocked-down GATA3 in human uncommitted T cell precursors, potential negative effects of GATA3 on the development of these cells were circumvented. Third, species-specific differences cannot be excluded, as demonstrated previously for instance with respect to the IL7 requirement during B cell development. Intriguingly, GATA3-mediated repression of human NK cell development may involve activation of *IL7R* expression as evident from GATA3 binding at that region, a feature that does not occur in mice. Consistently, T cell precursors from *IL2RG*-deficient patients fail to commit into the T-lineage pathway^48^, indicating that the stage of IL7R activation corresponds with the requirement for GATA3 to induce T-cell commitment. Although we did not find many differences in GATA3 occupancy between human and mouse, we may have missed other species-specific regulatory events that are controlled by GATA3 since we performed ChIP on total human thymocytes. Indeed, data in mouse show clear stage-specific changes in GATA3 occupancy^28^ that may be masked in our human results because of this approach. Further studies will be required to investigate this in more detail.

The capacity of GATA3 to repress alternative lymphoid differentiation, as well as stem cell genes such as *LMO2*, *MEF2C*, *TAL1*, *PU1* and *BCL11A* is consistent with observations in mice^10^. In this way, GATA3 function in human also substantially overlaps with that of BCL11B, although this gene remains to be studied in human. Our study shows that, in human, TCF1 function seems more dependent on Notch1 activity compared with in mice since no initiation of T cell development can be observed by TCF1 in the absence of Notch activation. This may relate to the high level of Notch activation that is required during this specification stage. Nevertheless, the core transcriptional components of the T-lineage network seems identical in human and mouse, although species-specific differences in kinetics and function are clear.

Given that Notch signalling is critical in various developmental systems, our findings help to understand the mechanisms through which Notch signalling induces and supports lineage choices in these different contexts. Following the initial Notch activation phase that involves the induction of both positive and negative downstream mediators, Notch signalling further depends on the activity of lineage-specific genes that downregulate the negative downstream mediators and, as such, this allows Notch to exercise its positive regulatory roles during the initiation of various developmental programmes. The combined activation of both positive and negative downstream mediators thereby provides a mechanistic framework through which Notch can maintain progenitor cells in a multipotent state. While Notch is critical to induce particular developmental programmes, such a dual system may form the molecular basis for understanding lateral inhibition and can permit alternative lineages to differentiate in case environmental conditions do not support further progression of the ‘default' programme.

## Methods

### Isolation of haematopoietic progenitor cells and thymocytes

Child thymus was obtained from children undergoing cardiac surgery with informed consent of the parents or guardian. Umbilical CB and peripheral blood samples were obtained with informed consent and all human samples were used according to the guidelines of the Medical Ethical Commission of Ghent University Hospital (Belgium). Within 24 h after collection, mononuclear cells were isolated via Lymphoprep density-gradient. CD34^+^ cells were purified by CD34 magnetic-activated cell-sorting (MACS) beads (Miltenyi Biotec), according to the manufacturer's instructions. Subsequently, enriched CD34^+^ cells were labelled with CD34-PE (Miltenyi Biotec, 130-081-002), CD3-APC (BD Biosciences, 345767), CD14-APC (Miltenyi Biotec, 130-091-243), CD19-APC (Miltenyi Biotec, 130-091-248) and CD56-APC (BD Biosciences, 130-091-248) according to the manufacturer's instructions, and CD34^+^Lin^−^ cells from CB were subsequently sorted with a FACSAriaII (BD Biosciences). CD34^+^ MACS purified thymocytes were stained with CD34-APC (BD Biosciences, 345804), CD1-FITC (clone OKT6, produced in-house and FITC labelled using Molecular Probes, F-1906) and CD4-PE (Miltenyi, 130-091-231) to sort CD34^+^CD1^−^CD4^−^ uncommitted and CD34^+^CD1^+^CD4^−^ committed early thymocytes. All in-house produced antibodies were used at 1 μg per 10^6^ cells. To obtain CD4^+^CD3^−^CD28^−^ and CD4^+^CD3^−^CD28^+^ cells, total thymus suspension was stained for 45 min with CD3 (clone OKT3, produced in-house and FITC labelled using Molecular Probes, F-1906), CD8 (clone OKT8, produced in-house and FITC labelled using Molecular Probes, F-1906) and Glycophorin (clone 10F7MN, produced in-house and used unlabelled) and antibody labelled cells were subsequently depleted using sheep anti mouse IgG magnetic Dynal beads (Invitrogen), according to the guidelines of the manufacturer. Next, the depleted cells were stained with CD4-PE, CD34-FITC (Miltenyi, 130-081-001), CD3-FITC (Miltenyi, 130-080-401), CD8-FITC (Miltenyi, 130-080-601) and CD28-APC (Miltenyi, 130-092-923) and CD4^+^CD34^−^CD3^−^CD8^−^CD28^−^ and CD4^+^CD34^−^CD3^−^CD8^−^CD28^+^ pre and post β-selected thymocytes were sorted. CD4^+^CD8^+^CD3^−^ and CD4^+^CD8^+^CD3^+^ double positive thymocytes where sorted following CD4-APC (Miltenyi, 130-091-232), CD8b-PECy7 (eBioscience, 25-5273-42) and CD3-FITC labelling of total thymus cell suspension^15^,^22^. Purity of the sorted cells was checked on a FACSCalibur or LSRII (BDIS) and was always >98%.

### Viral constructs

MISSION SHC002 (control shRNA), SHC1231 (*GATA3* shRNA), and SHC5022 and SHC5023 (*DTX1* shRNA) lentiviral vectors were purchased from Sigma in which the puromycin resistance gene was replaced with a PCR-amplified enhanced green fluorescent protein (EGFP) cDNA using BamHI and KpnI restriction sites. Validity of the constructs was confirmed by sequence analysis. Infectious lentivirus was produced by jetPEI (polyplus transfection)-mediated transfection of the 293 T-cell line with one of the MISSION vectors, in conjuction of the pCMV-VSV-G (envelope) and p8.91 (packaging) constructs. The virus supernatant was harvested 2 and 3 days after transfection and subsequently concentrated 10-times using PEG-it (System Biosciences), when necessary.

LZRS-GATA3-IRES-EGFP has been described^38^ and GATA3 cDNA from this vector was used to generate LZRS-GATA3-IRES-tNGFR by cloning it into LZRS-IRES-tNGFR (NGFR control). The cDNA encoding ROG was subcloned from the pCI-ROG plasmid^36^ into LZRS-IRES-EGFP (EGFP control) to generate the LZRS-ROG-IRES-EGP vector. TCF1 was subcloned from the MSCV-TCF1-EGFP (Weber) into LZRS-IRES-EGFP to generate LZRS-TCF1-IRES-EGFP. Recombinant virus from LZRS retroviral vectors was generated as described^22^,^38^.

### Coculture experiments

Sorted CD34^+^lin^−^ CB cells or CD34^+^ thymocytes were cultured in complete Iscove's Modified Dulbecco's Medium (IMDM) containing 10% FCS and supplemented with Thrombopoietin (TPO) (20 ng ml^−1^), stem cell factor (SCF) (100 ng ml^−1^) and FMS-like tyrosine kinase 3 ligand (FLT3-L) (100 ng ml^−1^) or SCF (10 ng ml^−1^) and interleukin (IL)7 (10 ng ml^−1^), respectively. One (thymocytes) or 2 days (CB CD34^+^ HPCs) later, cells were transduced with an equal volume of the appropriate retro- or lentiviruses using RetroNectin coated 24- or 96-well tissue culture plates to which additional cytokines were added to keep the concentration constant^38^. Forty-eight hours after transduction, cells were harvested and sorted for EGFP^+^ or NGFR^+^ transduced cells, in conjunction with human CD45 staining if derived from cocultures. In case of double transductions, cells were immediately infected with the second virus and cultured one additional day under the same conditions, before being cocultured the next day. In other cases, sorted cells were used for gene expression analysis, or immediate stromal cocultures in selected cultures conditions. OP9-GFP and OP9-DLL1 cocultures were performed in MEMα containing 20% FCS in the presence of FLT3-L, SCF and IL7 (all 5 ng ml^−1^). For the generation of NK cells on OP9 stromal cells, cocultures were additionally supplemented with 10 ng ml^−1^ IL15. Cocultures were performed in 24 wells and initiated with comparable cell numbers for each experimental setting within each experiment. Cell numbers for each condition are calculated for an input of 1,000 cells. At indicated time points, cells were harvested and analysed by flow cytometry.

### RNA extraction and quantitative reverse transcription PCR

RNA from sorted cell types, either directly derived from tissues or derived from 2-day OP9 cocultures in which human CD45 labelling was used to prevent OP9 contamination, was extracted using miRNeasy microkit (Qiagen) and converted into cDNA using Superscript RT II (Invitrogen). Real-time PCR reactions were performed using the LightCycler 480 SYBR Green I Master mix (Roche) on a LightCycler 480 real-time PCR system (Roche) using the following primers: HES1-Fw 5′-TGTCAACACGACACCGGATAAA-3′; HES1-Rev 5′-CCATAATAGGCTTTGATGACTTTCTG-3′; DTX1-Fw 5′-ACGAGAAAGGCCGGAAGGT-3′; DTX1-Rev 5′-GGTGTTGGACGTGCCGATAG-3′; NOTCH1-Fw 5′-GCAGTTGTGCTCCTGAAGAA-3′; NOTCH1-Rev 5′-CGGGCGGCCAGAAAC-3′; NOTCH3-Fw 5′-GTGATCGGCTCGGTAGTAATGC-3′; NOTCH3-Rev 5′-CTGACAACGCTCCCAGGTAGTC-3′; CD3E-Fw 5′-GGCAAGATGGTAATGAAGAAATGG-3′; CD3E-Rev 5′-AGGGCATGTCAATATTACTGTGGTT-3′; GATA3-5′-FwTGGGCTCTACTACAAGCTTCACAATAT-3′; GATA3-Rev 5′-TTGCTAGACATTTTTCGGTTTCTG-3′; RUNX3-Fw 5′-GGTGGCCAGGTTCAACGA-3′; RUNX3-Rev 5′-TGATGGTCAGGGTGAAACTCTTC-3′; IL7RA-Fw 5′-GGAGAAAGTGGCTATGCTCAAAA-3′; IL7RA-Rev 5′-TCCATTCACTTCCAACTGGCTAT-3′; TCF7-Fw 5′-CCCAACTCTCTCTCTACGAACATTT-3′; TCF7-Rev 5′-TGCAGAGGCCTGTGAACTTG-3′; MYC-Fw 5′-CGTCTCCACACATCAGCACAA-3′; MYC-Rev 5′-CACTGTCCAACTTGACCCTCTTG-3′; BCL11B-Fw 5′-GCAGCACTTGTCCCAGAG-3′; BCL11B-Rev 5′-CCACAGGTGAGCAGGTCA-3′; TCF12-Fw 5′-GGCGGCAGGACCTGCTA-3′; TCF12-Rev 5′-AGGTCGCTCAGCTCCTTGTC-3′; TCF12alt-Fw 5′-TCAACATGTATTGTGCTTATCCTGTC-3′; TCF12alt-Rev 5′-ACTGGTTGGTGGCTTAGGAGAT-3′; ID2-Fw 5′-TCAAGGACACAGGTGAAAGGT-3′; ID2-Rev 5′-CTGCAAGGACAGGATGCTGATA-3′. Primers for MITF (qHsaCED0037870), CPA3 (qHsaCED0046132), TPSAB1 (qHsaCED0034189), TAL1 (qHsaCID0013881) and GATA2 (qHsaCID0010528) were purchased from BioRad. Relative expression levels were calculated for each gene using *ACTB* or *GAPDH* for normalisation, as indicated.

### Gene expression analysis

RNA samples from control and *GATA3*-transduced CD34^+^ thymocytes, or from control shRNA, *GATA3* shRNA and *TCF7* shRNA transduced RPMI-8402 cells (purchased from DSMZ) were profiled on a custom designed Agilent microarray covering all protein coding genes (33,128 mRNA probes, Human Sureprint G3 8 × 60k micro-arrays (Agilent)) and 12,000 lncRNAs (23,042 unique lncRNA probes)^29^. For each transduction, three independent samples were profiled from three different thymus donors. Expression data were normalized using the VSN-package (Bioconductor release 2.12) in R. Differential expression analysis was performed in R using limma. All gene expression profiling data has been deposited in the GEO database (GSE71753). Public datasets (GSE29181, GSE22601 and GSE21774) were normalized using the Affy-package (Bioconductor release 2.12) in R. NK cell signatures were generated by selecting the top 500 significant differentially expressed genes in human NK cell subsets^49^ compared with human committed T cell precursors^50^. A Notch-dependent gene list was generated by selecting the top 500 most significantly upregulated genes in CD34^+^ human thymocytes that were cultured on OP9-DLL1 versus on OP9-GFP (ref. 29).

### Chromatin immunoprecipitation and high-throughput sequencing

Two independent ChIP experiments were performed on 50 million total thymocytes with 2.5 μg GATA-3 antibody (sc-268, Santa Cruz Biotechnology), using an established protocol^28^. ChIP samples were processed as described^28^, and sequenced using Illumina Genome Analyzer II. Reads were mapped to the hg19 reference genome using Bowtie2 using default settings. Peak calling was performed with MACS2 using input samples as control. Motif enrichment was done with MEME-ChIP using 500 bp regions centred on the peak summits as defined by MACS2. BED files were converted to fasta files using BEDtools. Peak distribution around transcriptional start site was determined using GREAT. ChIP data have been deposited in the GEO database (GSE71753).

### Statistical analysis

A non-parametric paired Wilcoxon test or paired Student's *t*-test was used as indicated and a *P* value of <0.05 was considered statistically significant. For microarray data, adjusted *P* values for multiple testing were calculated using the limma package in R to control false discovery rate and an adjusted *P* value of <0.05 was considered statistically significant.

## Additional information

**How to cite this article:** Van de Walle, I. *et al*. GATA3 induces human T cell commitment by restraining Notch activity and repressing NK-cell fate. *Nat. Commun.* 7:11171 doi: 10.1038/ncomms11171 (2016).

## Supplementary Material

Supplementary Figures and Supplementary Reference Supplementary Figures 1-12 and Supplementary Reference

Supplementary Data 1 Full micro-array data from control and GATA3 transduced CD34^+^ thymocytes, 48 hours following transduction. Data shows average of 3 independent experiments

Supplementary Data 2 Full micro-array data from *GATA3* versus control shRNA transduced RPMI-8402 cells, 48 hours following transduction. Data shows average of 2 independent experiments.

Supplementary Data 3 Full micro-array data from *TCF7* versus *GATA3* shRNA transduced RPMI-8402 cells, 48 hours following transduction. Data shows average of 2 independent experiments.

Supplementary Data 4 Analysis of GATA3 binding (using GATA3 ChIP-seq data from total human thymocytes) for human CD56^bright^ NK cell-signature genes.

Supplementary Data 5 Analysis of GATA3 binding (using GATA3 ChIP-seq data from total human thymocytes) for human CD56^dim^ NK cell-signature genes.

Supplementary Data 6 Analysis of Notch1 binding (using Notch1 ChIP-seq data from CUTLL1 cells, Wang et al) and Notch dependent expression (using RNAseq data from OP9-GFP versus OP9-DLL1 cultured human CD34^+^ thymocytes, Durinck et al) for human CD56^bright^ NK-cell signature genes

Supplementary Data 7 Analysis of Notch dependent expression (using RNAseq data from OP9-GFP versus OP9-DLL1 cultured human CD34^+^ thymocytes, Durinck et al), Notch1 binding (using Notch1 ChIP-seq data from CUTLL1 cells, Wang et al) and GATA3 binding (using GATA3 ChIP-seq data from total human thymocytes) for genes significantly downregulated at the CD34^+^CD1- to CD34^+^CD1^+^ transition that marks human T-lineage commitment (Dik et al)

## References

[b1] Taghon T. Cell Determination during Hematopoiesis (eds Brown G., Ceredig R. 141–177Nova (2009).

[b2] Taghon T., Waegemans E. & Van de Walle I. Notch signaling during human T cell development. Curr. Top. Microbiol. Immunol. 360, 75–97 (2012). 22692833 10.1007/82_2012_230

[b3] De Obaldia M. E. & Bhandoola A. Transcriptional regulation of innate and adaptive lymphocyte lineages. Annu. Rev. Immunol. 33, 607–642 (2015). 25665079 10.1146/annurev-immunol-032414-112032

[b4] Rothenberg E. V. Transcriptional control of early T and B cell developmental choices. Annu. Rev. Immunol. 32, 283–321 (2014). 24471430 10.1146/annurev-immunol-032712-100024 PMC3994230

[b5] Radtke F. . Deficient T cell fate specification in mice with an induced inactivation of Notch1. Immunity 10, 547–558 (1999). 10367900 10.1016/s1074-7613(00)80054-0

[b6] Schmitt T. M., Ciofani M., Petrie H. T. & Zuniga-Pflucker J. C. Maintenance of T cell specification and differentiation requires recurrent notch receptor-ligand interactions. J. Exp. Med. 200, 469–479 (2004). 15314075 10.1084/jem.20040394 PMC2211933

[b7] Taghon T. N., David E. S., Zuniga-Pflucker J. C. & Rothenberg E. V. Delayed, asynchronous, and reversible T-lineage specification induced by Notch/Delta signaling. Genes Dev. 19, 965–978 (2005). 15833919 10.1101/gad.1298305 PMC1080135

[b8] Weber B. N. . A critical role for TCF-1 in T-lineage specification and differentiation. Nature 476, 63–68 (2011). 21814277 10.1038/nature10279 PMC3156435

[b9] Germar K. . T-cell factor 1 is a gatekeeper for T-cell specification in response to Notch signaling. Proc. Natl Acad. Sci. USA 108, 20060–20065 (2011). 22109558 10.1073/pnas.1110230108 PMC3250146

[b10] Garcia-Ojeda M. E. . GATA-3 promotes T-cell specification by repressing B-cell potential in pro-T cells in mice. Blood 121, 1749–1759 (2013). 23287858 10.1182/blood-2012-06-440065

[b11] Scripture-Adams D. D. . GATA-3 dose-dependent checkpoints in early T cell commitment. J. Immunol. 193, 3470–3491 (2014). 25172496 10.4049/jimmunol.1301663 PMC4170028

[b12] Ikawa T. . An essential developmental checkpoint for production of the T cell lineage. Science 329, 93–96 (2010). 20595615 10.1126/science.1188995

[b13] Li L., Leid M. & Rothenberg E. V. An early T cell lineage commitment checkpoint dependent on the transcription factor Bcl11b. Science 329, 89–93 (2010). 20595614 10.1126/science.1188989 PMC2935300

[b14] Li P. . Reprogramming of T cells to natural killer-like cells upon Bcl11b deletion. Science 329, 85–89 (2010). 20538915 10.1126/science.1188063 PMC3628452

[b15] Van de Walle I. . An early decrease in Notch activation is required for human TCR-alphabeta lineage differentiation at the expense of TCR-gammadelta T cells. Blood 113, 2988–2998 (2009). 19056690 10.1182/blood-2008-06-164871

[b16] Waegemans E. . Notch3 activation is sufficient but not required for inducing human T-lineage specification. J. Immunol. 193, 5997–6004 (2014). 25381438 10.4049/jimmunol.1400764

[b17] De Smedt M. . Active form of Notch imposes T cell fate in human progenitor cells. J. Immunol. 169, 3021–3029 (2002). 12218117 10.4049/jimmunol.169.6.3021

[b18] Garcia-Peydro M., de Yebenes V. & Toribio M. L. Sustained Notch1 signaling instructs the earliest human intrathymic precursors to adopt a gammadelta T-cell fate in fetal thymus organ culture. Blood 102, 2444–2451 (2003). 12829602 10.1182/blood-2002-10-3261

[b19] Van de Walle I. . Specific Notch receptor-ligand interactions control human TCR-alphabeta/gammadelta development by inducing differential Notch signal strength. J. Exp. Med. 210, 683–697 (2013). 23530123 10.1084/jem.20121798 PMC3620353

[b20] De Smedt M. . Notch signaling induces cytoplasmic CD3 epsilon expression in human differentiating NK cells. Blood 110, 2696–2703 (2007). 17630354 10.1182/blood-2007-03-082206

[b21] Weerkamp F. . Identification of Notch target genes in uncommitted T-cell progenitors: No direct induction of a T-cell specific gene program. Leukemia 20, 1967–1977 (2006). 16990763 10.1038/sj.leu.2404396

[b22] Taghon T. . Notch signaling is required for proliferation but not for differentiation at a well-defined beta-selection checkpoint during human T-cell development. Blood 113, 3254–3263 (2009). 18948571 10.1182/blood-2008-07-168906

[b23] Ikawa T., Kawamoto H., Goldrath A. W. & Murre C. E proteins and Notch signaling cooperate to promote T cell lineage specification and commitment. J. Exp. Med. 203, 1329–1342 (2006). 16682500 10.1084/jem.20060268 PMC2121213

[b24] Mingueneau M. . The transcriptional landscape of alphabeta T cell differentiation. Nat. Immunol. 14, 619–632 (2013). 23644507 10.1038/ni.2590 PMC3660436

[b25] Van de Walle I. . Jagged2 acts as a Delta-like Notch ligand during early hematopoietic cell fate decisions. Blood 117, 4449–4459 (2011). 21372153 10.1182/blood-2010-06-290049 PMC3673751

[b26] Hozumi K. . Notch signaling is necessary for GATA3 function in the initiation of T cell development. Eur. J. Immunol. 38, 977–985 (2008). 18383037 10.1002/eji.200737688

[b27] Taghon T., Yui M. A. & Rothenberg E. V. Mast cell lineage diversion of T lineage precursors by the essential T cell transcription factor GATA-3. Nat. Immunol. 8, 845–855 (2007). 17603486 10.1038/ni1486 PMC3140173

[b28] Zhang J. A., Mortazavi A., Williams B. A., Wold B. J. & Rothenberg E. V. Dynamic transformations of genome-wide epigenetic marking and transcriptional control establish T cell identity. Cell 149, 467–482 (2012). 22500808 10.1016/j.cell.2012.01.056 PMC3336965

[b29] Durinck K. . The Notch driven long non-coding RNA repertoire in T-cell acute lymphoblastic leukemia. Haematologica 99, 1808–1816 (2014). 25344525 10.3324/haematol.2014.115683 PMC4258754

[b30] Xu W. & Kee B. L. Growth factor independent 1B (Gfi1b) is an E2A target gene that modulates Gata3 in T-cell lymphomas. Blood 109, 4406–4414 (2007). 17272506 10.1182/blood-2006-08-043331

[b31] Sanda T. . Core transcriptional regulatory circuit controlled by the TAL1 complex in human T cell acute lymphoblastic leukemia. Cancer Cell 22, 209–221 (2012). 22897851 10.1016/j.ccr.2012.06.007 PMC3422504

[b32] Braunstein M. & Anderson M. K. HEB in the spotlight: Transcriptional regulation of T-cell specification, commitment, and developmental plasticity. Clin. Dev. Immunol. 2012, 678705 (2012). 22577461 10.1155/2012/678705 PMC3346973

[b33] Homminga I. . Integrated transcript and genome analyses reveal NKX2-1 and MEF2C as potential oncogenes in T cell acute lymphoblastic leukemia. Cancer Cell 19, 484–497 (2011). 21481790 10.1016/j.ccr.2011.02.008

[b34] Anderson M. K. . Definition of regulatory network elements for T cell development by perturbation analysis with PU.1 and GATA-3. Dev. Biol. 246, 103–121 (2002). 12027437 10.1006/dbio.2002.0674

[b35] Yokota Y. . Development of peripheral lymphoid organs and natural killer cells depends on the helix-loop-helix inhibitor Id2. Nature 397, 702–706 (1999). 10067894 10.1038/17812

[b36] Miaw S. C., Choi A., Yu E., Kishikawa H. & Ho I. C. ROG, repressor of GATA, regulates the expression of cytokine genes. Immunity 12, 323–333 (2000). 10755619 10.1016/s1074-7613(00)80185-5

[b37] Wang H. . Genome-wide analysis reveals conserved and divergent features of Notch1/RBPJ binding in human and murine T-lymphoblastic leukemia cells. Proc. Natl Acad. Sci. USA 108, 14908–14913 (2011). 21737748 10.1073/pnas.1109023108 PMC3169118

[b38] Taghon T. . Enforced expression of GATA-3 severely reduces human thymic cellularity. J. Immunol. 167, 4468–4475 (2001). 11591773 10.4049/jimmunol.167.8.4468

[b39] Ciofani M. & Zuniga-Pflucker J. C. Notch promotes survival of pre-T cells at the beta-selection checkpoint by regulating cellular metabolism. Nat. Immunol. 6, 881–888 (2005). 16056227 10.1038/ni1234

[b40] Garbe A. I. & von Boehmer H. TCR and Notch synergize in alphabeta versus gammadelta lineage choice. Trends Immunol. 28, 124–131 (2007). 17261380 10.1016/j.it.2007.01.004

[b41] Taghon T., Yui M. A., Pant R., Diamond R. A. & Rothenberg E. V. Developmental and molecular characterization of emerging beta- and gammadelta-selected pre-T cells in the adult mouse thymus. Immunity 24, 53–64 (2006). 16413923 10.1016/j.immuni.2005.11.012

[b42] De Obaldia M. E. . T cell development requires constraint of the myeloid regulator C/EBP-alpha by the Notch target and transcriptional repressor Hes1. Nat. Immunol. 14, 1277–1284 (2013). 24185616 10.1038/ni.2760 PMC4038953

[b43] Douglas N. C., Jacobs H., Bothwell A. L. & Hayday A. C. Defining the specific physiological requirements for c-Myc in T cell development. Nat. Immunol. 2, 307–315 (2001). 11276201 10.1038/86308

[b44] Izon D. J. . Deltex1 redirects lymphoid progenitors to the B cell lineage by antagonizing Notch1. Immunity 16, 231–243 (2002). 11869684 10.1016/s1074-7613(02)00271-6

[b45] Zhang J. . The genetic basis of early T-cell precursor acute lymphoblastic leukaemia. Nature 481, 157–163 (2012). 22237106 10.1038/nature10725 PMC3267575

[b46] Vosshenrich C. A. . A thymic pathway of mouse natural killer cell development characterized by expression of GATA-3 and CD127. Nat. Immunol. 7, 1217–1224 (2006). 17013389 10.1038/ni1395

[b47] Freud A. G., Yu J. & Caligiuri M. A. Human natural killer cell development in secondary lymphoid tissues. Semin. Immunol. 26, 132–137 (2014). 24661538 10.1016/j.smim.2014.02.008 PMC4010312

[b48] Six E. M. . Cytokines and culture medium have a major impact on human in vitro T-cell differentiation. Blood Cells Mol. Dis. 47, 72–78 (2011). 21531153 10.1016/j.bcmd.2011.04.001

[b49] Juelke K. . CD62L expression identifies a unique subset of polyfunctional CD56dim NK cells. Blood 116, 1299–1307 (2010). 20505160 10.1182/blood-2009-11-253286

[b50] Dik W. A. . New insights on human T cell development by quantitative T cell receptor gene rearrangement studies and gene expression profiling. J. Exp. Med. 201, 1715–1723 (2005). 15928199 10.1084/jem.20042524 PMC2213269

