# Supplementary material for: GATA3 induces human T-cell commitment by restraining Notch activity and repressing NK-cell fate
Source: Nat Commun. 2016 Apr 6;7:11171. doi: 10.1038/ncomms11171 (PMC4823830; doi:10.1038/ncomms11171)
Supplement: Supplementary Figures and Supplementary Reference — Supplementary Figures 1-12 and Supplementary Reference [file ncomms11171-s1.pdf]

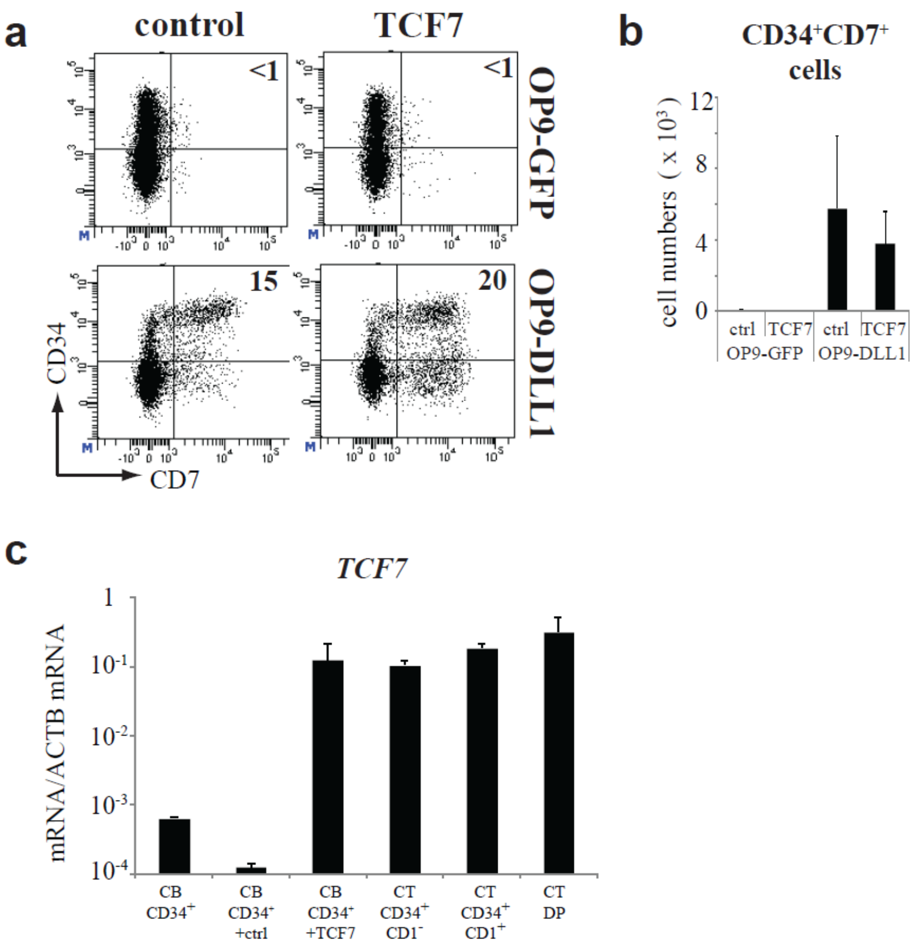

Supplementary Figure 1. TCF1 by itself fails to induce human T-lineage specification

(**a**) Flow cytometry analysis of control and *TCF7* transduced cord blood CD34$^+$lin$^-$ precursors in OP9-GFP and OP9-DLL1 co-cultures in the presence of IL7, SCF and FLT3L, showing the development of CD34$^+$CD7^{++} early T cell precursors after 6 days of co-culture. (**b**) Absolute numbers of CD34$^+$CD7^{++} T precursor cells developed in corresponding cultures from **a**. Data shows average of 3 independent experiments and error bars indicate SEM. (**c**) Quantitative PCR for *TCF7* expression in freshly isolated CB CD34$^+$ HPCs, in control and *TCF7* transduced CB CD34$^+$ HPCs and in ex vivo isolated intra-thymic CD34$^+$CD1$^-$ uncommitted, CD34$^+$CD1$^+$ T-lineage committed and DP thymocytes. Data shows the average expression of 2-4 independent experiments, relative to *ACTB* levels and error bars indicate SEM.

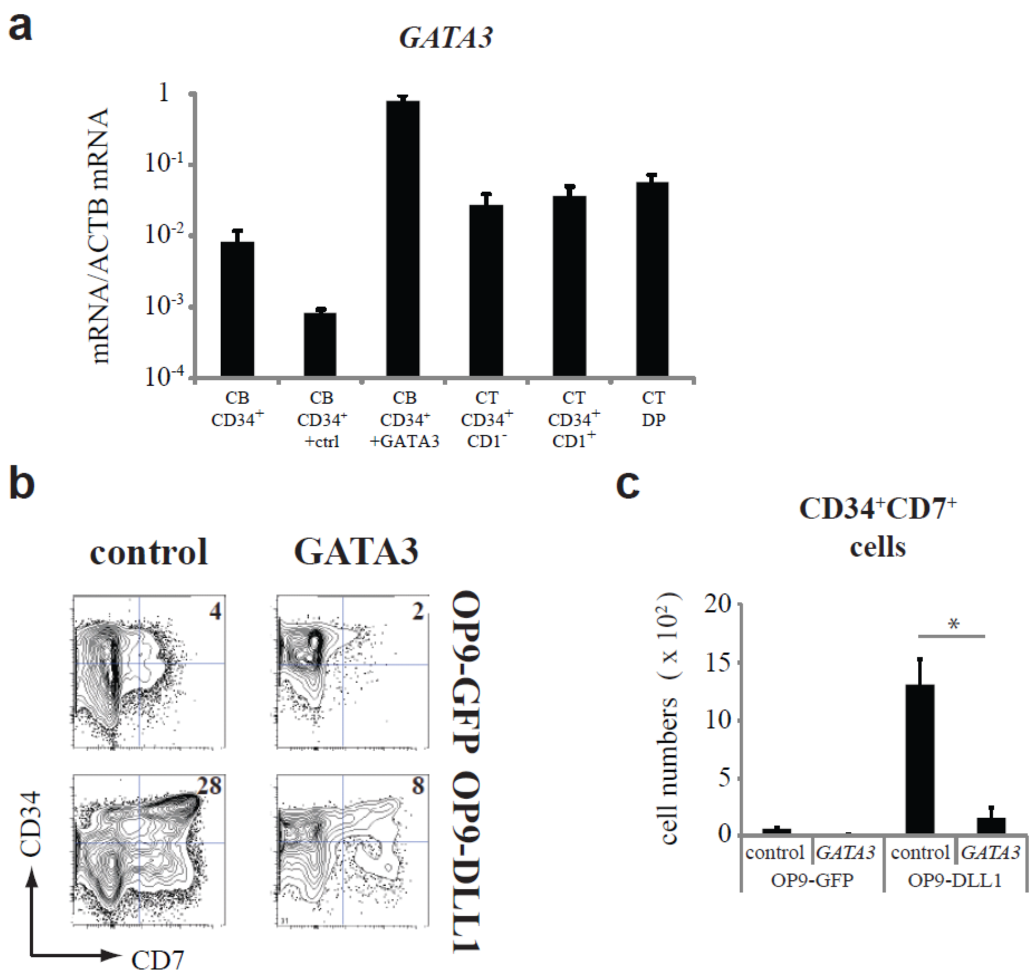

Supplementary Figure 2. GATA-3 inhibits Notch-induced T cell specification.

(**a**) Quantitative PCR for *GATA3* expression in freshly isolated CB CD34$^+$ HPCs, in control and *GATA3* transduced CB CD34$^+$ HPCs and in ex vivo isolated intra-thymic CD34$^+$CD1$^-$ uncommitted, CD34$^+$CD1$^+$ T-lineage committed and DP thymocytes. Data shows the average expression of 2-4 independent experiments, relative to *ACTB* levels and error bars indicate SEM. (**b**) Flow cytometry analysis of control and *GATA3* transduced cord blood CD34$^+$lin$^-$ precursors in OP9-GFP and OP9-DLL1 co-cultures in the presence of IL7, SCF and FLT3L, showing the development of CD34$^+$CD7^{++} early T cell precursors after 6 days of co-culture. (**c**) Absolute numbers of CD34$^+$CD7^{++} T precursor cells developed in corresponding cultures from **b**. Data shows the average of 7 independent experiments and error bars indicate SEM. * $P < 0.05$ (non-parametric paired Wilcoxon test)

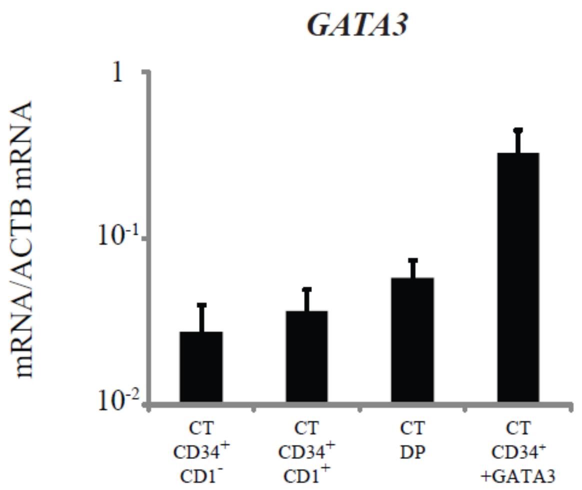

Supplementary Figure 3. GATA3 expression levels in *in vivo* and in transduced human thymocytes

Quantitative PCR for *GATA3* expression in ex vivo isolated intra-thymic $CD34^+CD1^-$ uncommitted, $CD34^+CD1^+$ T-lineage committed, DP thymocytes and *GATA3* transduced $CD34^+$ thymocytes. Data shows the average expression of 2-4 independent experiments, relative to *ACTB* levels and error bars indicate SEM.

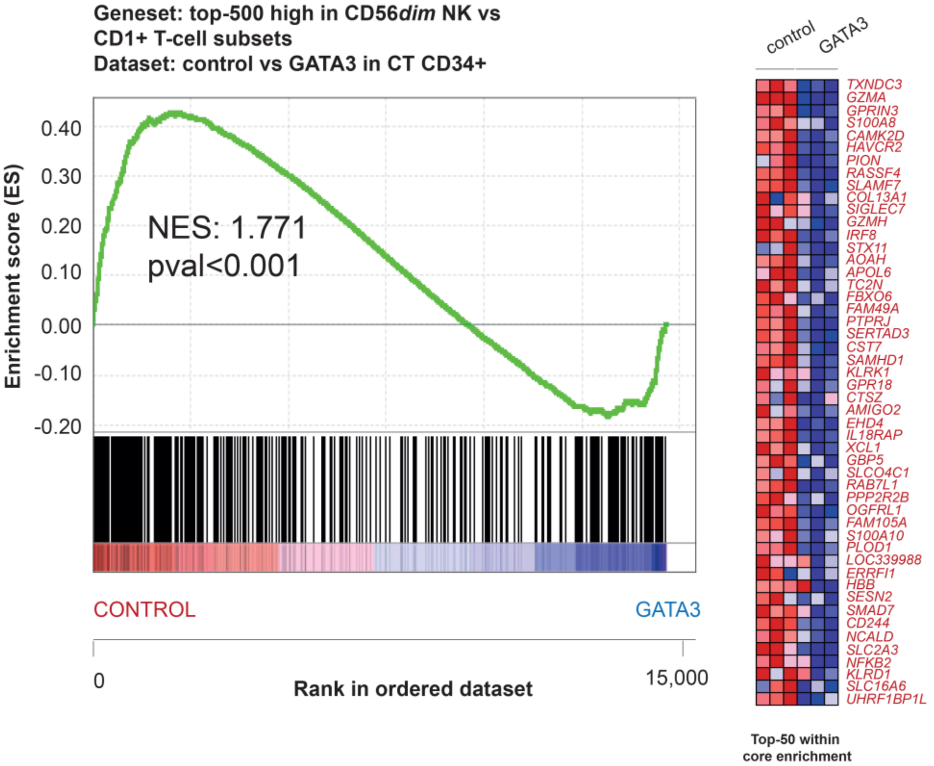

Supplementary Figure 4. GATA3 inhibits expression of NK-cell signature genes

GSEA shows a significant enrichment for CD56^{bright} human NK cells signature genes in control versus GATA3 transduced human CD34^{+} thymocytes.

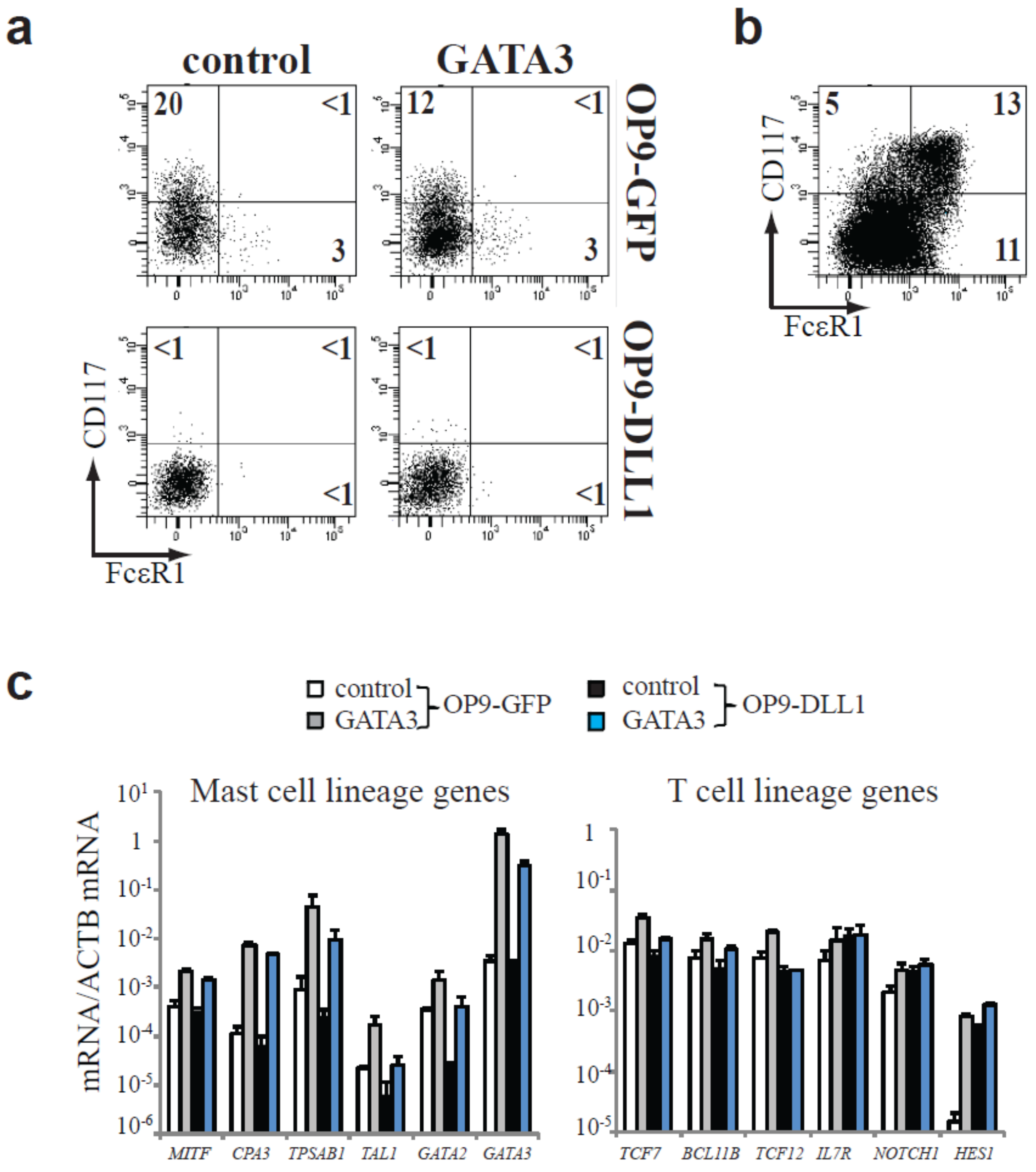

Supplementary Figure 5. GATA3 overexpression does not induce mast cell development.

(**a**) Flow cytometry analysis of CD117 versus FcεR1 staining in control and *GATA3* transduced $CD34^{+}CD1^{-}$ uncommitted thymocytes in OP9-GFP and OP9-DLL1 co-cultures. Data is representative for 3 independent experiments. (**b**) Flow cytometry analysis of $CD117^{+}FcεR1^{+}$ mast cells derived from $CD34^{+}$ CB HPCs after 19 days of culture. (**c**) Quantitative PCR analysis of changes of gene expression in control (white and black bars) and GATA3 (grey and blue bars) transduced $CD34^{+}$ thymocytes after 2 day co-culture on OP9-GFP (white and grey bars) or OP9-DLL1 (black and blue bars). Data show the average expression of 2 independent experiments, relative to ACTB levels. Error bars indicate SEM.

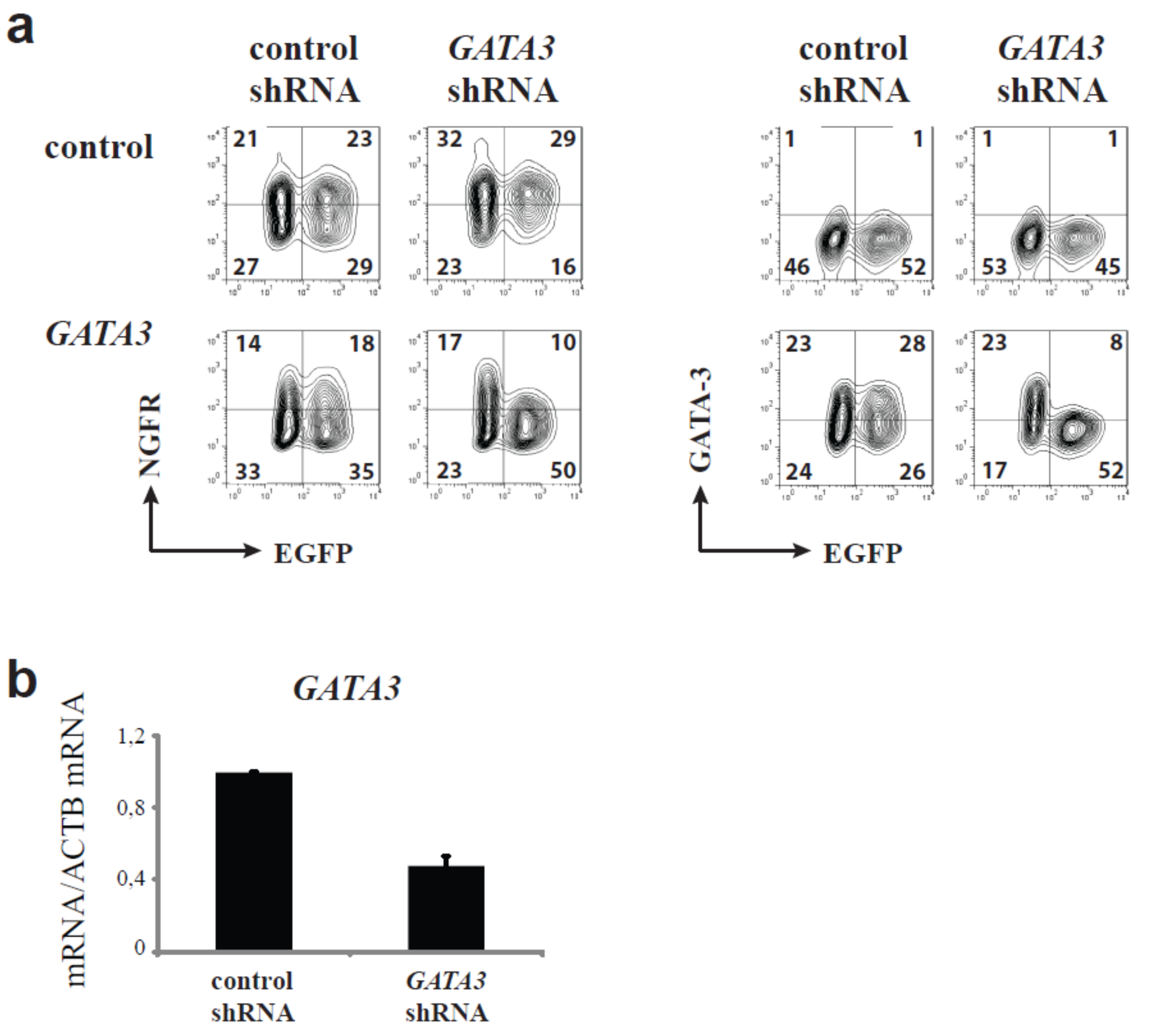

Supplementary Figure 6. GATA3 directed shRNA reduces GATA3 protein and mRNA levels

(**a**) Flow cytometry analysis of HL60 cells, transduced with a control-IRES-NGFR or *GATA3*-IRES-NGFR retroviral vector, combined with a EGFP encoding control shRNA or *GATA3* shRNA lentiviral vectors, showing NGFR versus EGFP (left) or intracellular GATA-3 versus EGFP (right) expression. Both NGFR and *GATA3* expression are reduced in case of *GATA3* shRNA-mediated knockdown, indicating that the shRNA enhances degradation of the bicistronic mRNA. (**b**) Quantitative PCR for *GATA3* expression in control shRNA and *GATA3* shRNA transduced CD34$^+$ thymocytes, 48 hours after transduction. Data shows the average expression of 3 independent experiments, relative to *ACTB* levels and relative to control shRNA transduced cells. Error bars indicate SEM.

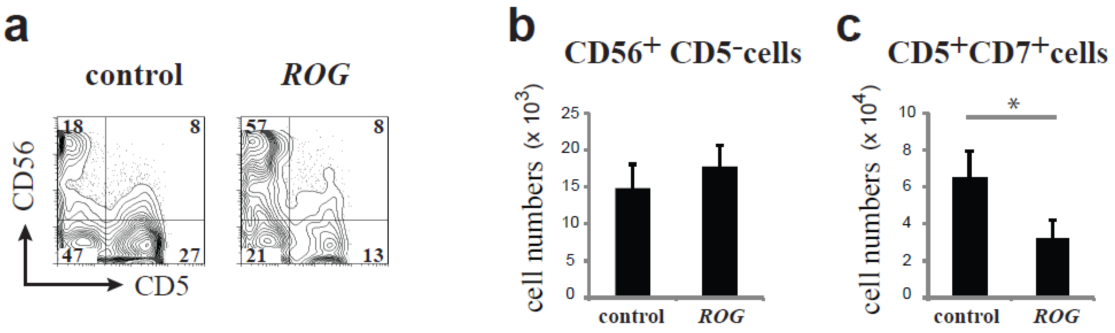

Supplementary Figure 7. ROG overexpression confirms differential GATA3 requirement for T and NK cell development .

(**a**) Flow cytometry analysis of control and *ROG* transduced $CD34^+$ CB progenitors in 2-week OP9-DLL1 co-cultures in the presence of IL7, SCF, FLT3L and IL15. Graphs show absolute number of $CD56^+$ $CD5^-$ NK cells (**b**) and $CD5^+CD7^+$ T cells (**c**) that developed in corresponding cultures from a. Data shows the average of 5 independent experiments and error bars indicate SEM. * $P < 0.05$ (non-parametric paired Wilcoxon test)

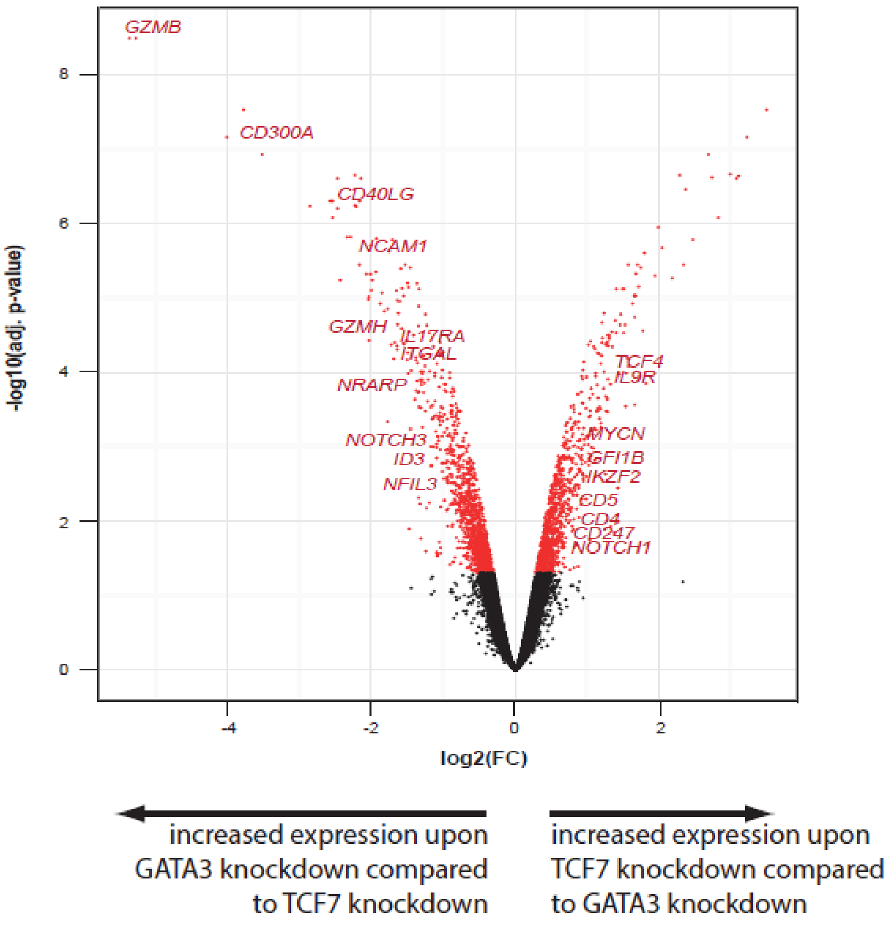

Supplementary Figure 8. GATA3 and TCF7 have unique regulatory roles.

Volcano plot showing genes with significant differential expression between TCF7 and GATA3 shRNA transduced RPMI-8402 cells. Data shows significant differential expressed genes over 2 independent experiments. Red dots represent the significant differentially expressed genes (adjusted *P*-value <0.05).

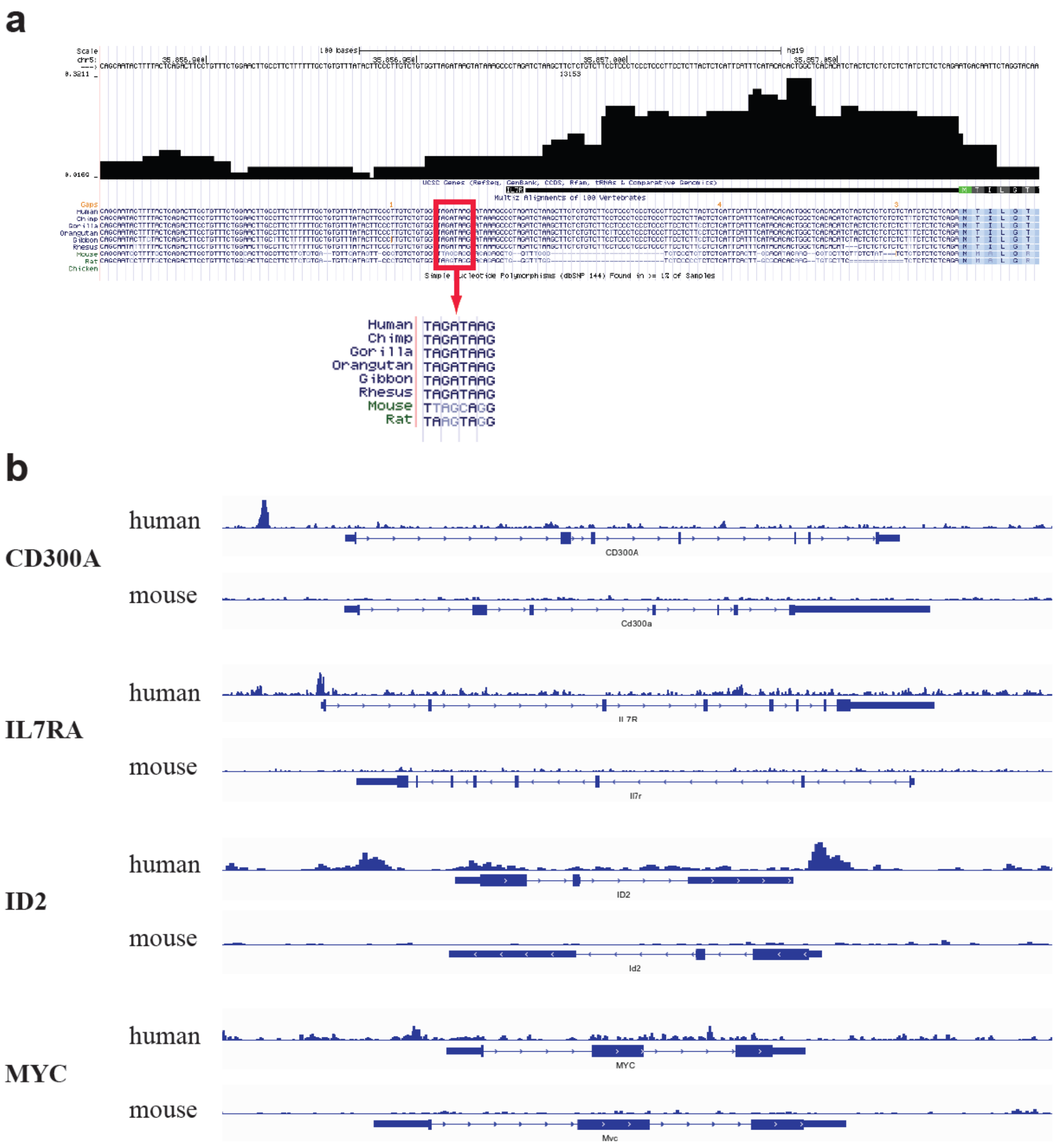

Supplementary Figure 9. GATA3 occupancy across species

(**a**) UCSC genome browser view of GATA3 binding at the human IL7R locus, showing conservation of the GATA binding site in primates, but not rodents. (**b**) GATA3 binding at selected gene loci in human total thymocytes versus mouse DN2b thymocytes[1].

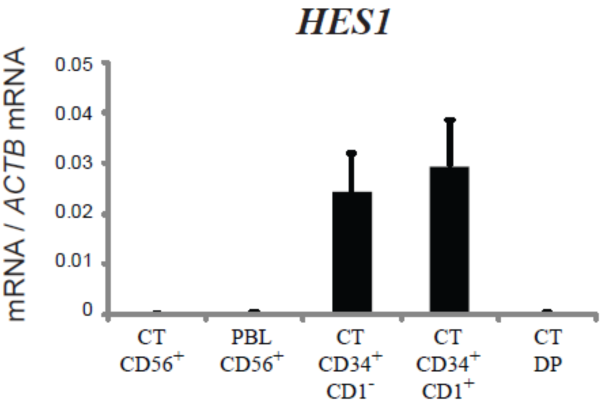

Supplementary Figure 10. Thymic NK cells do not show global signs of Notch activation

Quantitative PCR for *HES1* expression in thymus (CT) and peripheral blood (PBL) CD56$^+$ NK cells, compared to intra-thymic CD34$^+$CD1$^-$ uncommitted and CD34$^+$CD1$^+$ committed progenitors and DP thymocytes. Data shows average expression, relative to *ACTB*, of 2-5 independent samples and error bars indicate SEM.

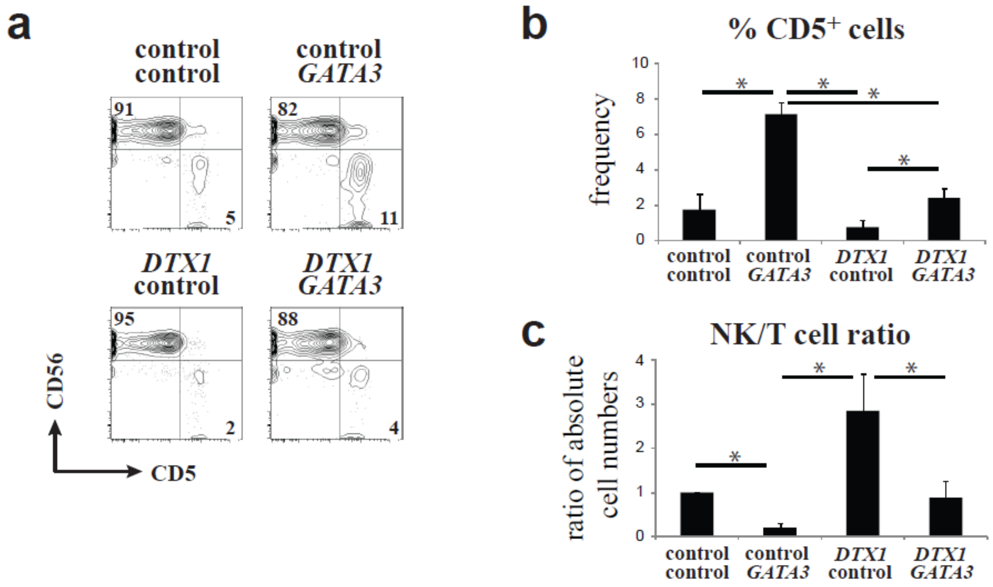

Supplementary Figure 11. The balance between DTX1 and GATA3 levels determines intrathymic NK vs T cell development.

(**a**) Flow cytometry analysis of control-control, control-*GATA3*, *DTX1*-control and *DTX1*-*GATA3* double transduced CD34$^+$CD1$^-$ uncommitted thymocytes in 2-week OP9-DLL1 co-cultures in the presence of IL7, SCF, FLT3L and IL15. (**b**) Average frequency of CD5$^+$ T-lineage cells from cultures in **a**. (**c**) NK/T cell ratio from cultures depicted in **a**. Data shows the average of 4 independent experiments and error bars indicate SEM. * $P < 0.05$ (paired t-test)

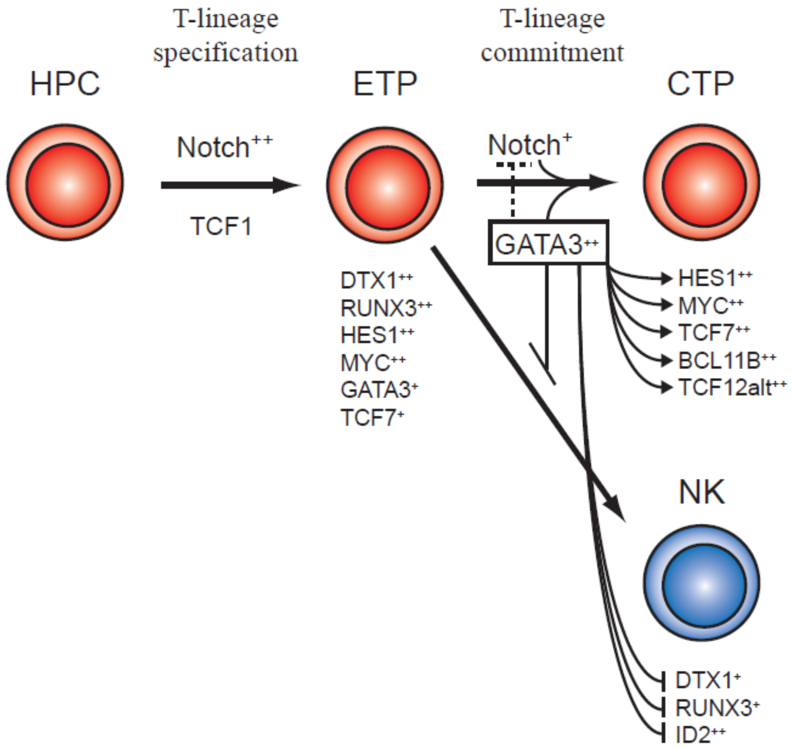

Supplementary Figure 12. Molecular regulation of human T cell commitment.

Schematic overview of the molecular processes that control early T cell development in human, with focus on GATA3-mediated effects on the induction of human T cell commitment at the expense of NK cell development. HPC: hematopoietic precursor cell; ETP: early T-cell precursor; CTP: committed T-cell precursor. Dotted line indicates partial inhibition.

Supplementary Reference

1. Zhang,J.A., Mortazavi,A., Williams,B.A., Wold,B.J., & Rothenberg,E.V. Dynamic transformations of genome-wide epigenetic marking and transcriptional control establish T cell identity. *Cell* **149**, 467-482 (2012).
